# Current Practices in LC-MS Untargeted Metabolomics: A Scoping Review on the Use of Pooled Quality Control Samples

**DOI:** 10.1021/acs.analchem.3c02924

**Published:** 2023-12-06

**Authors:** Corey D. Broeckling, Richard D. Beger, Leo L. Cheng, Raquel Cumeras, Daniel J. Cuthbertson, Surendra Dasari, W. Clay Davis, Warwick B. Dunn, Anne Marie Evans, Alvaro Fernández-Ochoa, Helen Gika, Royston Goodacre, Kelli D. Goodman, Goncalo J. Gouveia, Ping-Ching Hsu, Jennifer A. Kirwan, Dritan Kodra, Julia Kuligowski, Renny Shang-Lun Lan, María Eugenia Monge, Laura W. Moussa, Sindhu G. Nair, Nichole Reisdorph, Stacy D. Sherrod, Candice Ulmer Holland, Dajana Vuckovic, Li-Rong Yu, Bo Zhang, Georgios Theodoridis, Jonathan D. Mosley

**Affiliations:** † Analytical Resources Core: Bioanalysis and Omics Center; Department of Agricultural Biology, Colorado State University, Fort Collins, Colorado 80525, United States; ‡ Division of Systems Biology, National Center for Toxicological Research, U.S. Food and Drug Administration, Jefferson, Arkansas 72079, United States; § Departments of Radiology and Pathology, Massachusetts General Hospital, Harvard Medical School, Boston, Massachusetts 02114, United States; ∥ Department of Oncology, Hospital Universitari Sant Joan de Reus, Institut d’Investigació Sanitària Pere Virgili (IISPV), URV, CERCA, 43204 Reus, Spain; ⊥ Agilent Technologies Inc., 5301 Stevens Creek Blvd, Santa Clara, California 95051, United States; # Department of Quantitative Health Sciences, Mayo Clinic, Rochester, Minnesota 55905, United States; ● National Institute of Standards and Technology, Chemical Sciences Division, 331 Fort Johnson Road, Charleston, South Carolina 29412, United States; ◑ Centre for Metabolomics Research, Department of Biochemistry and Systems Biology, Institute of Systems, Molecular and Integrative Biology, University of Liverpool, BioSciences Building, Crown St., Liverpool L69 7ZB,U.K.; ◐ Metabolon, Inc. 617 Davis Drive, Suite 100, Morrisville, North Carolina 27560, United States; ○ Department of Analytical Chemistry, University of Granada, 18071 Granada, Spain; ◒ Metabolon, Inc., 617 Davis Drive, Suite 100, Morrisville, North Carolina 27560, United States; ◓ School of Medicine, Aristotle University of Thessaloniki, 54124 Thessaloniki, Greece; ⦶ Institute for Bioscience and Biotechnology Research, National Institute of Standards and Technology, University of Maryland, Gudelsky Drive, Rockville, Maryland 20850, United States; ⊕ Department of Environmental Health Sciences, University of Arkansas for Medical Sciences, Little Rock, Arkansas 72205-7190, United States; ◊ Metabolomics, Berlin Institute of Health at Charite, Anna-Louisa-Karsch-Str. 2, 10178 Berlin, Germany; ⧫ Department of Chemistry, Aristotle University of Thessaloniki, 54124 Thessaloniki, Greece; ⬡ Neonatal Research Group, Health Research Institute La Fe, Avenida Fernando Abril Martorell 106, 46026 Valencia, Spain; ⬢ Arkansas Children’s Nutrition Center, Little Rock, Arkansas 72202-3591, United States; ⬠ Centro de Investigaciones en Bionanociencias (CIBION), Consejo Nacional de Investigaciones Científicas y Técnicas (CONICET), Godoy Cruz 2390, C1425FQD Ciudad de Buenos Aires, Argentina; □ Center for Veterinary Medicine, Office of New Animal Drug Evaluation, U.S. Food and Drug Administration, Rockville, Maryland 20855, United States; ■ Department of Biological Sciences, University of Alberta, Edmonton, AB T6G 2G2, Canada; ⊡ Department of Pharmaceutical Sciences, University of Colorado−Anschutz Medical Campus, Aurora, Colorado 80045, United States; ◨ Department of Chemistry and Center for Innovative Technology, Vanderbilt University, Nashville, Tennessee 37240, United States; ◧ Chemistry Branch, Eastern Laboratory, Office of Public Health Science, USDA-FSIS, Athens, Georgia 30605, United States; ⬒ Department of Chemistry and Biochemistry, Concordia University, 7141 Sherbrooke Street West, Montreal, QC H4B 1R6, Canada; ⬓ Division of Systems Biology, National Center for Toxicological Research, U.S. Food and Drug Administration, Jefferson, Arkansas 72079, United States; ⬕ Olaris, Inc., 175 Crossing Blvd Suite 410, Framingham, Massachusetts 01702, United States; ◪ Department of Chemistry, Aristotle University of Thessaloniki, Thessaloniki 54124, Greece; ◩ Center for Environmental Measurement and Modeling, Environmental Protection Agency, Athens, Georgia 30605, United States

## Abstract

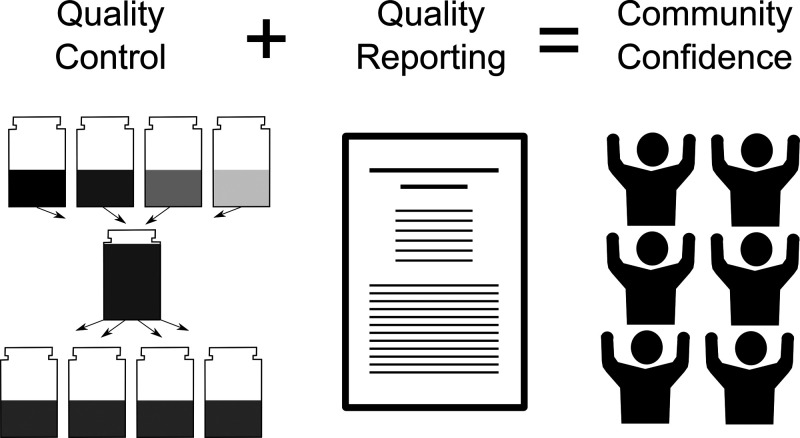

Untargeted metabolomics is an analytical approach with numerous applications serving as an effective metabolic phenotyping platform to characterize small molecules within a biological system. Data quality can be challenging to evaluate and demonstrate in metabolomics experiments. This has driven the use of pooled quality control (QC) samples for monitoring and, if necessary, correcting for analytical variance introduced during sample preparation and data acquisition stages. Described herein is a scoping literature review detailing the use of pooled QC samples in published untargeted liquid chromatography–mass spectrometry (LC-MS) based metabolomics studies. A literature query was performed, the list of papers was filtered, and suitable articles were randomly sampled. In total, 109 papers were each reviewed by at least five reviewers, answering predefined questions surrounding the use of pooled quality control samples. The results of the review indicate that use of pooled QC samples has been relatively widely adopted by the metabolomics community and that it is used at a similar frequency across biological taxa and sample types in both small- and large-scale studies. However, while many studies generated and analyzed pooled QC samples, relatively few reported the use of pooled QC samples to improve data quality. This demonstrates a clear opportunity for the field to more frequently utilize pooled QC samples for quality reporting, feature filtering, analytical drift correction, and metabolite annotation. Additionally, our survey approach enabled us to assess the ambiguity in the reporting of the methods used to describe the generation and use of pooled QC samples. This analysis indicates that many details of the QC framework are missing or unclear, limiting the reader’s ability to determine which QC steps have been taken. Collectively, these results capture the current state of pooled QC sample usage and highlight existing strengths and deficiencies as they are applied in untargeted LC-MS metabolomics.

## Introduction

Untargeted metabolomics has been shown to be an effective technical platform for characterizing the small molecules within a sample, both qualitatively and using relative quantitation. Metabolomics approaches are increasingly applied to human health, agriculture, biotechnology, ecology, environmental sciences, toxicology, microbiology, synthetic biology, and regulatory science. Due to its high sensitivity, specificity, and broad detection capabilities, liquid chromatography coupled to high-resolution mass spectrometry (LC-HRMS, or hereafter LC-MS for the sake of brevity) is one of the most widely employed techniques for untargeted metabolomics. These characteristics make LC-MS appealing, but LC-MS is also susceptible to batch effects and background interferences. Quality control (QC) processes are especially challenging for untargeted analytical approaches, including metabolomics.^1,2^ Coordinated efforts to emphasize quality management measures have emerged in recent years. However, quality management protocols in the field of metabolomics have been slow to gain community-wide acceptance, in part due to a lack of standardization as it relates to sample type, MS ionization conditions, LC mobile phase solvent composition, column chemistry, and gradient, among other experimental conditions. Of note, other analytical approaches are also used in metabolomics, and while gas chromatography (GC) is generally more standardized than liquid chromatography, many of the same concerns related to quality management apply to GC-MS and other hyphenated MS approaches.

The use of pooled QC samples (also considered a specific type of “intrastudy QC”^3^) and other types of QC samples (e.g., blank samples, internal standards, standard reference materials) have been adopted with varying frequency by the metabolomics community.^4−8^ A pooled QC sample, which is generated by pooling aliquots of the study samples, can be considered the “average” of all samples.^6,9,10^ The pooled QC sample can be combined prior to sample extraction and extracted using the same sample preparation protocol(s) as those employed for the study samples. Alternatively, the pooled QC sample can be prepared by combining aliquots of the study sample extracts after sample preparation has been performed prior to LC-MS analysis. While not equivalent, these approaches are both categorized as pooled QC samples. Pooled QC samples are then analyzed alongside the study samples periodically throughout the injection series. The pooled QC sample approach derives from the “fit-for-purpose” targeted chemical methods in which the technical performance of an analytical method is independently validated based on a simulated sample with related physical and chemical properties comparable to the test samples.^11^ Such QC practice has been conducted in regulated manufacturing areas and in clinical assays. In untargeted chemical assays, the pooled QC sample is most frequently used as an assay control derived from experimental samples, which may be used to describe and correct for variance but is not a part of any of the final statistical experimental design.

There are several potential uses and limitations of a pooled QC sample.^12,13^ One of the primary roles of pooled QC samples is to assess variability in sample preparation and/or instrument performance.^14,15^ Pooled QC samples provide an untargeted and feature-specific estimate of the analytical repeatability and reproducibility of metabolite measurements. The pooled QC can also be used as an initial assessment of system suitability prior to a study or for analytical system conditioning. Since the pooled QC is an “average” sample, it may also be used to support in-depth annotation efforts. Dilutions series of QC samples have been used in a manner analogous to calibration curves to confirm response linearity.^16^ Finally, pooled QC samples can be used to assess and correct for intra- and inter-batch technical variation and monitor long-term intralab precision, enabling integration from multiple analytical batches,^3,17^ which has enabled large-scale studies (where *n* > 1000) through correction of unavoidable technical variance over several months to years.^18,19^ The most notable weaknesses of the pooled QC sample approach are that (1) relatively infrequently detected features can be diluted to undetectable levels, and if a feature is not detected in the pooled QC sample, it cannot be used to report the quality of that feature or correct experimental data; (2) the qualitative and quantitative composition of the sample is uncharacterized, preventing its use in supporting absolute quantitative goals; and (3) every intrastudy pooled QC sample will be unique, limiting its use in aligning data sets across laboratories or studies. Alternate (or additional) QC approaches, such as the inclusion of isotopically labeled internal standards, interstudy (long-term) pooled QC samples or standardized reference materials can be used to complement these weaknesses.^6^

The frequency with which papers report the use of a pooled QC sample approach in metabolomics has been increasing as a result of increased education and prior calls for metabolomics scientists to use them as part of their QC processes.^6^ The pooled QC sample approach enables recurrent injections of an “average” sample for that sample set, thereby enabling analytical assay precision to be calculated for untargeted metabolomic experiments. It is therefore of great value to understand how pooled QC samples are currently being employed in LC-MS-based metabolomics studies and to highlight their utility.

Herein, we describe the results of a rigorous scoping^20,21^ review of recently published LC-MS-based untargeted metabolomics studies to (1) describe the frequency and ways in which pooled QC samples are used and (2) document the frequency of ambiguous reporting of how pooled QC samples are prepared and used. Additionally, we briefly document the types of alternative QC samples used together or in lieu of pooled QC samples to guide future efforts toward understanding metabolomic QC approaches.

## Methods

### Literature Search

This survey was designed to review recent untargeted publicly available and indexed metabolomics studies utilizing liquid chromatography coupled to mass spectrometry (LC-MS), which aimed to compare two or more sample groups using relative quantification. Web of Science was queried on April 4, 2022 using the following search string: (METABONOMIC* OR (METABOL* PROFIL*) OR (METABOL* PHENOTYPING*) OR METABOTYP* OR (METABOL* FINGERPRINT*) OR (METABOL* SIGNATURE*) OR (METABOL* RESPONSE*) OR (METABOL* PERTURBATION*) OR (PROFIL* OF METABOLITES*) OR (PROFIL* OF ENDOGEN* METABOLITE*) OR METABOLOME) AND (LCMS OR LC/MS OR (MASS SPECTROMETRY*) OR (LIQUID CHROMATOGRAPHY*) OR HPLC-MS OR UHPLC-MS OR UPLC-MS OR (ULTRA PERFORMANCE*) OR TOF MS OR UNTARGETED* OR TOF-MS OR ORBITRAP OR HRMS OR LC-TOF OR NON-TARGETED* OR Q-TOF-MS OR LC-HR-MS), with the data range restricted to January 1 to July 1, 2021. The full complement of references meeting these criteria were selected (*n* = 721 papers). The articles were assigned a random order using the “sample” function in R.

**Figure 1:**
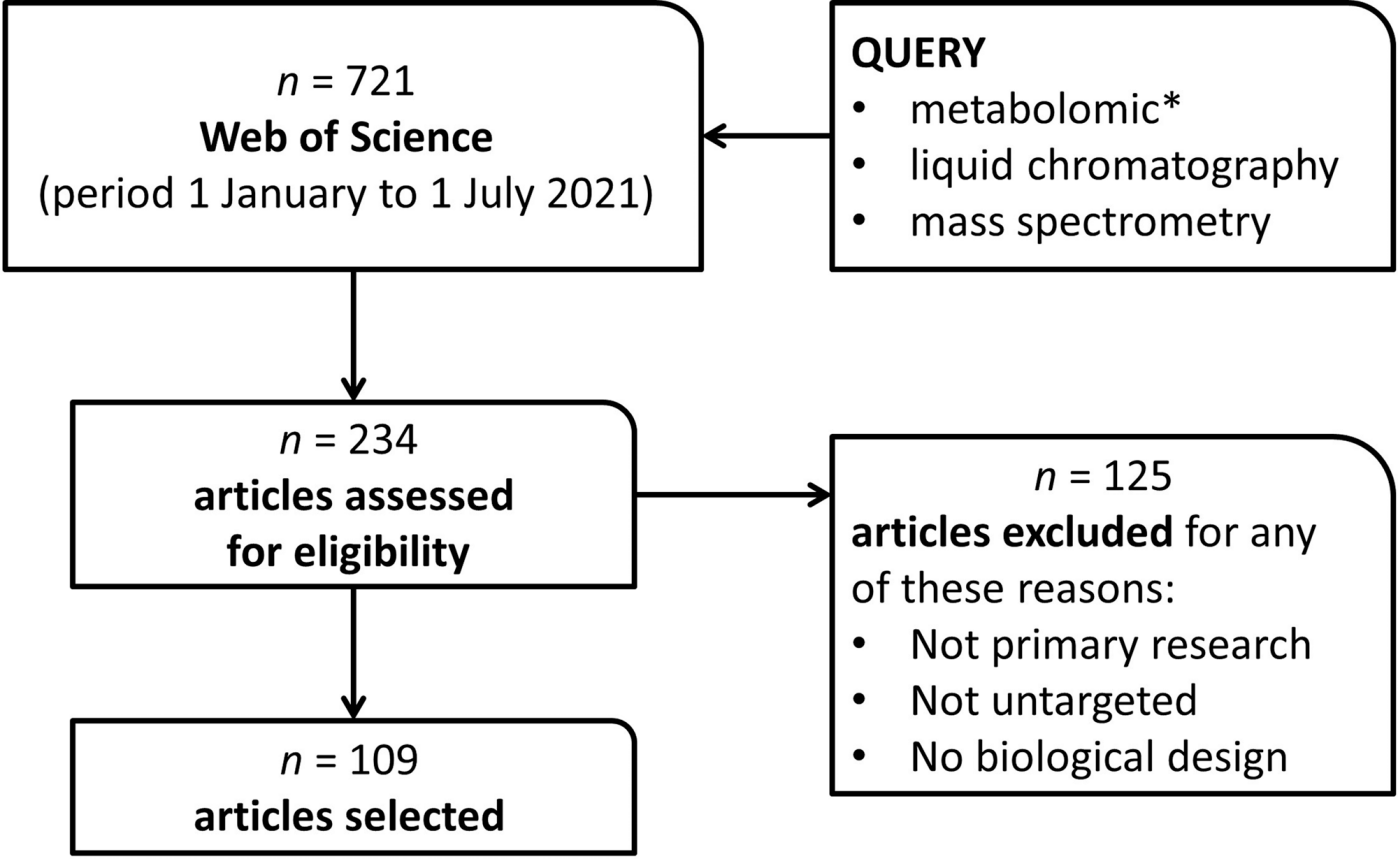
Literature search, screening, and selection workflow. 721 papers were returned from the Web of Science query, and 234 papers were screened to enable the selection of 109 papers for review. * indicates a wildcard character.

Review papers, purely analytically focused methodological articles, and studies that did not use an untargeted LC-MS-based approach were eliminated from consideration (Figure 1). Specifically, review papers were those that did not report new data and, therefore, had no methods section present. Analytical methodology-focused experimental designs were eliminated from consideration. For example, a paper describing the optimization of chromatographic separation or extraction conditions was not considered relevant to the scope of the review. Finally, the methods were reviewed manually to confirm the use of LC-MS untargeted data acquisition approaches. Reviewers were instructed to use both the methods in the main body of the paper and the online Supporting Information, but not to pursue referenced literature. To capture referenced literature which may offer additional method details, the survey asked a question whether the paper cited references when describing their QC procedures. To have each paper reviewed at least five times and considering the number of reviewers available (31) each reviewing ∼20 papers, a target goal of reviewing 110 papers was selected. Papers were manually evaluated (in randomized order) based on the exclusion criteria above. Each paper was either selected or removed from consideration until the target of 110 selected papers was met. Specifically, 234 papers were manually screened to enable selection of these 110 papers that report primary research results using untargeted LC-MS based metabolomics to explore a nonanalytical, comparative experimental design (Figure 1). No criterion for the type of study (biological, environmental, clinical, etc.) was used in selecting papers. During the formal review, one additional paper was excluded due to the use of targeted acquisition methods, resulting in a final 109 papers surveyed.

Finally, all scoping reviewers were randomly assigned (“sample” function in R) approximately 20 different papers such that each of the 110 papers was assigned to five or more reviewers. Care was taken so that no reviewer was assigned a paper of which they were listed as a coauthor. The full list of papers reviewed can be found in the Supporting Information (supplemental_1_literature.surveyed.csv). The randomized selection process ensured that the surveyed literature is broadly representative of the full complement of Web of Science indexed peer-reviewed literature from this time period.

### Survey Structure

The survey was built using Google Forms, and the full survey question list is provided as supplemental_2_fullSurvey.pdf. The initial draft of the survey was refined following a pilot study, where five randomly selected papers were reviewed by all 31 reviewers. The results of this survey were only used to enable constructive feedback to improve the survey, which included both adding and removing questions as well as refining questions when the wording was unclear. For the final survey, some questions were only visible conditional on answers provided in prior questions to enable more detailed follow-up questions. For example, if the reviewer answered that a pooled QC approach was not used, they were not prompted to answer questions about how the pooled QC sample was used. Responses were collected over a six week period, and the results were exported to .csv format for further analysis.

### Data Curation and Analysis

The responses for each paper by reviewer were minimally curated to enable a more efficient summary of the results. The reviewer’s name, assigned paper, and date/time stamp were evaluated to remove duplicate reviews. If two reviews on the same paper were submitted by the same reviewer, the later submission (by date and time stamp) was retained. Some sample types were difficult to classify and were manually curated posthoc for consistency in reporting. Specifically, “propolis”, a product of honeybee pollen collection, was classified as “plant”, and “human cell lines” were classified as “mammalian” samples. The final curated data set representing each individual survey response is supplied as supplemental_3_individual.responses.csv.

All subsequent data processing was performed in R (v 4.1.2). The full R markdown^20^ script used is supplied as supplemental_4.txt, the output of which provides a full description of the results for every question (supplemental_5_details.pdf). If the reviewers were asked but failed to answer a question, this resulted in an empty cell value in the exported .csv file. These were classified as “no response” (“nr”). Empty cells were also derived from the conditional survey structure. For example, if the reviewer reported that pooled QC samples were not used, the reviewer would not see questions asking about the specific use(s) of pooled QC. In this case, for example, the reviewer would have a “no response” value for a subsequent question asking whether pooled QC samples were used in Principal Component Analysis (PCA), resulting in an empty cell. These were also coded as “nr”. No attempt was made to distinguish between these two values. Assignment of “nr” values was performed at the individual review level, prior to determination of the consensus answer for each paper.

Reviewer concordance was reported by examining the range of answers provided by the reviewers for each paper/question combination. A “concordant” answer across reviewer responses was defined as responses for which at least two-thirds of the reviewers provided the same response to a specific question. When the threshold for concordance was not met, the answer was assigned as “discordant”. For some questions, “Unclear” was offered as an answer in the question. These responses were reported separately to “discordant” in all plots/tables unless otherwise noted.

## Results

### Study Overview

In total, each of the 109 papers was reviewed by at least five reviewers, answering up to 46 questions related to the study design and the use of QC samples and processes. Of these, 67 papers performed metabolomics studies on samples derived from mammalian systems, 7 from nonmammalian animals (i.e., insects, reptiles, etc.), 30 from plants, and 10 from microbes; some papers performed analysis on samples from more than one taxon. The majority of papers (86) analyzed one type of sample, while 14 papers analyzed two or more sample types. Nine papers were assigned as discordant for the sample type count, indicating that the five reviewers did not agree on how many sample types were analyzed. Seventy-six papers analyzed sample sets comprising 1–50 independent samples, 18 papers analyzed 51–200 independent samples, 5 papers analyzed 201–1000 independent samples, and 10 papers were assigned as discordant for sample number.

### Pooled QC Sample Usage Frequency

**Figure 2:**
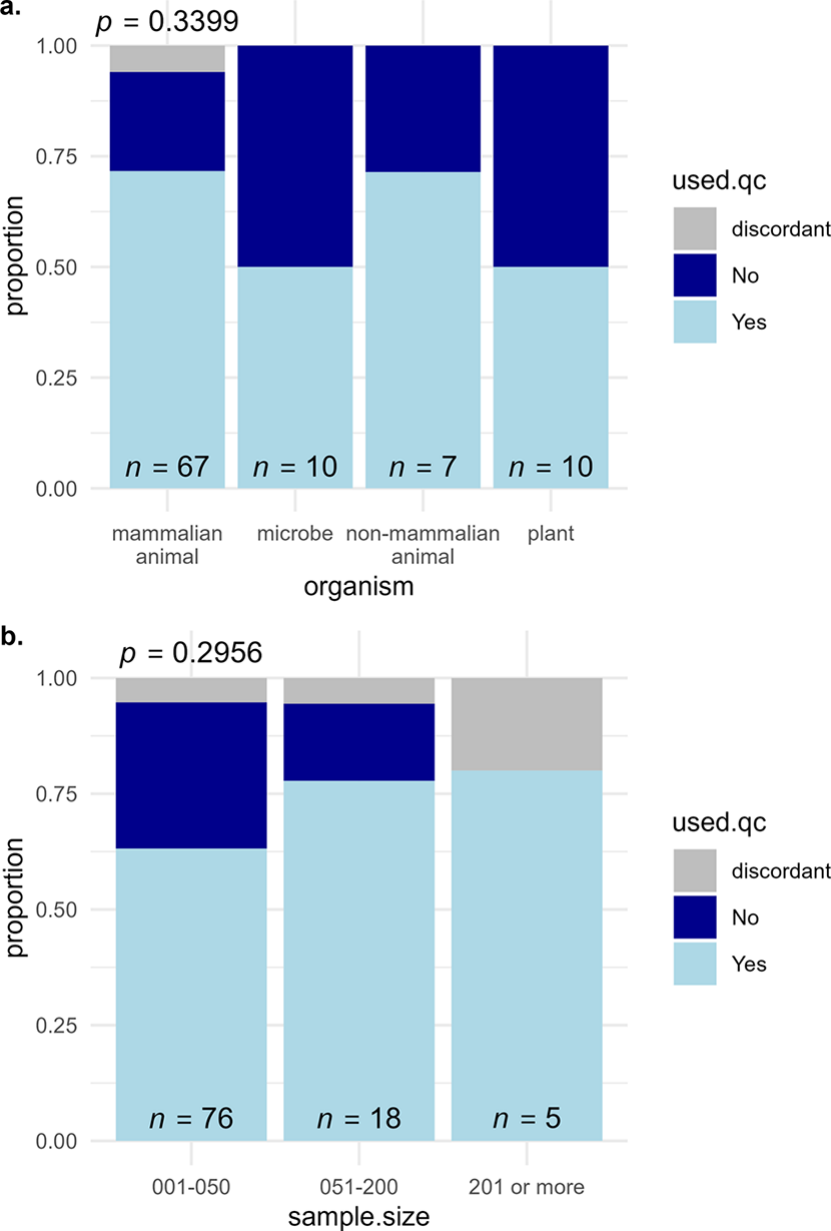
Proportion of studies that report usage of pooled QC samples by (a) organism class studied or (b) study sample size. Plot headers represent χ^2^ testing *p*-values testing the null hypothesis in equal proportions across categories.

Of 109 papers surveyed, 72 reported using a pooled QC sample in the study, 31 did not use a pooled QC sample, and 6 were assigned as discordant. Figure 2a displays these data by organism class. While there was some level of variance across organism classes in the proportion of studies that reported the use of pooled QC samples, χ^2^ testing revealed no significant differences between classes, indicating that pooled QC sample usage is reported to have been used at similar frequencies across biological taxa. A trend was observed toward higher reporting of pooled QC samples for larger studies (Figure 2b), but this trend was also not significant with χ^2^ testing. Since the survey’s primary focus was the use of pooled QC, the six discordant results for this question were further investigated. Among the six papers, one study used a pooled QC sample and described its preparation, so there was no clear reason for observed discordance. The remaining five studies reveal some of the ambiguities in reporting of the use of a pooled QC sample in the literature. One study apparently used a pooled QC sample but did not describe its preparation, so it was unclear if the sample used was indeed a pooled QC. One study did not describe the use of a pooled QC sample but cited another manuscript for further information. Three studies reported use of a pooled QC sample in a manner which did not fall into any survey answer categories. For these three studies, a pooled QC sample was used for targeted lipid identification only, for “additional monitoring”, or for optimizing injection volume. None of these three studies used pooled QC for QC of their untargeted metabolomics study.

Of the papers that reported pooled QC sample usage, 34 papers analyzed solid samples/matrices such as tissues, food, or dried blood spots, and 37 described the analysis of liquid samples such as plasma, urine, or surface water. Two papers analyzed both solid and liquid matrices, and three papers were assigned a consensus answer of “discordant”.

### Pooled QC Sample Creation

The vast majority of studies that utilized pooled QC samples, 56 out of 72 papers, generated a pooled QC sample from all biological samples in the sample set. Two papers generated the pooled QC sample from a subset of biological samples, and an additional four and ten papers were classified as “unknown” or “discordant”, respectively. The two papers that generated a pooled QC sample from a subset of all biological samples represented larger studies with sample sizes of 51–200 samples and 201–1000. While this was not stated by the authors, this suggests that the use of a subset of samples was driven by practical issues, such as available technician time, sample availability, or sample stability concerns.

QC samples in studies of solid samples can be generated by pooling sample material directly, by pooling extracts derived from each sample, or by pooling reconstituted extracts after the extract was dried. Papers were generally unclear regarding which option was used. Of 34 papers reporting studies on solid samples, 19 papers were classified as discordant, 7 as unclear, 4 reported to pool solid samples prior to extraction, and 4 reported to pool extracts after extracting solid samples.

Similarly, QC samples can be generated before or after extraction when liquid samples are utilized. For studies focused on liquid samples (*n* = 37), 7 papers were “unclear”, 15 were “discordant”, 14 generated QC samples by pooling directly from the biofluid, and 1 pooled after sample preparation. Note that the generation of a pooled QC sample prior to sample preparation allows for the pooled QC sample variance to account for the collective variance of sample preparation and analytical variance, while the generation of a pooled QC sample after sample preparation accounts for only analytical variance. The pooled QC approach generally does not allow for isolating the sample preparation from analytical variance.

### Pooled QC Samples Usage

**Figure 3:**
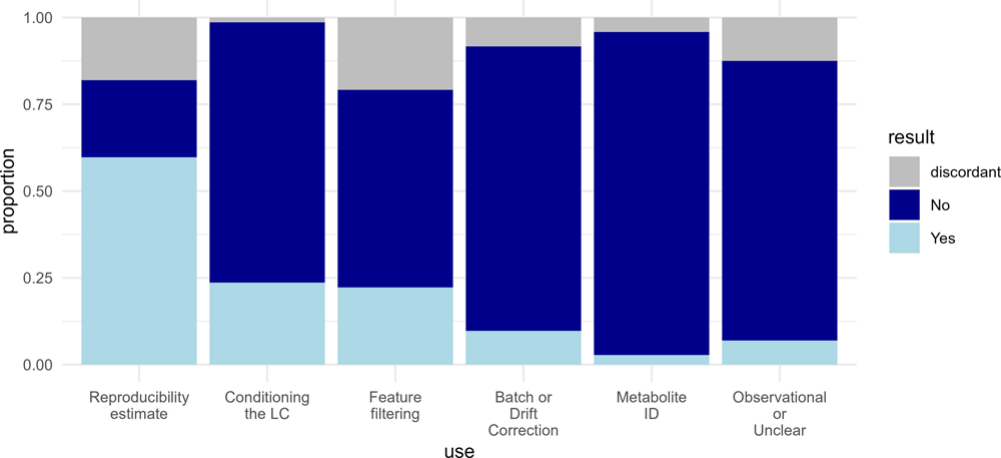
Pooled QC usage across 72 articles which were reported using a pooled QC approach.

Pooled QC samples can serve many functions. The reported uses for the pooled QC sample injections varied across papers (Figure 3). Sixty percent of the papers surveyed used the pooled QC sample to estimate repeatability/reproducibility, 24% for conditioning the LC system, 22% for filtering low-quality features, 10% for batch or drift correction, and 3% for supporting metabolite ID. Seven percent of papers were reported as having no clear indication that the QC samples were used for any of the above. Discordance frequency was quite high for these questions, particularly for reproducibility estimates and feature filtering, indicating that reviewers frequently had different interpretations on how the QC samples had been used in the study.

Of the 72 articles that reported the use of a pooled QC sample, 53 injected the pooled QC sample multiple times, while 8 papers were reported as “unclear”, and 11 as “discordant”. Replicate injections could be made from either a single vial or multiple vials; in only one of these 53 articles was it explicitly clear to *all* reviewers that the injection was made from multiple vials. In 40 articles, the consensus answer was assigned as “unclear”, and another 12 were found to be described as “discordant”. It was frequently difficult to assess where in the sample run order the pooled QC samples were injected: “beginning of batch” = 21 true, 18 false, and 14 discordant; “end of batch” = 10 true, 18 false, and 25 discordant; “middle of batch” = 39 true, 7 false, and 7 discordant. The majority of papers injected pooled QC samples every 6–10 injections (29 of 53), followed by every 2–5 injections (10 of 53) and every >10 injections (2 of 53). Eight papers were listed as “unclear” and four as “discordant” with respect to injection frequency.

Incorporation of pooled QC samples in a PCA scores plot is one approach by which to visually demonstrate relative reproducibility. When used in this manner, pooled QC samples are analyzed with the experimental samples by principal components analysis. The scores plot variance in the pooled QC samples represents analytical variance, and the variance of the full sample set represents biological variance. High data quality is demonstrated by showing relatively low pooled QC (analytical) variance as compared to sample (biological) variance. Thirty-one of 72 of the papers that used a pooled QC sample approach reported the use of pooled QC samples in a PCA, nearly all of which (30 of 31) plotted the pooled QC samples with the full biological sample set. When PCA plots were used to evaluate the QC, only one paper was reported to have used a quantitative metric describing pooled QC variance relative to sample variance, whereas the rest used visual inspection only.

A dilution series of pooled QC samples can also be used to demonstrate linearity in detector response, enabling filtering to remove features that fail to respond linearly with changes in concentration. Of 109 reviewed papers, none (*n* = 0) were reported to have clearly used a dilution series of the pooled QC sample in their QC regime, with two “discordant” responses noted.

When papers reported using multiple sample types (*n* = 12), reviewers generally reported that the pooled QC sample approach was mostly the same among the various matrices, with six “yes”, two “no”, and four “discordant” responses.

### Other QC Approaches

**Figure 4:**
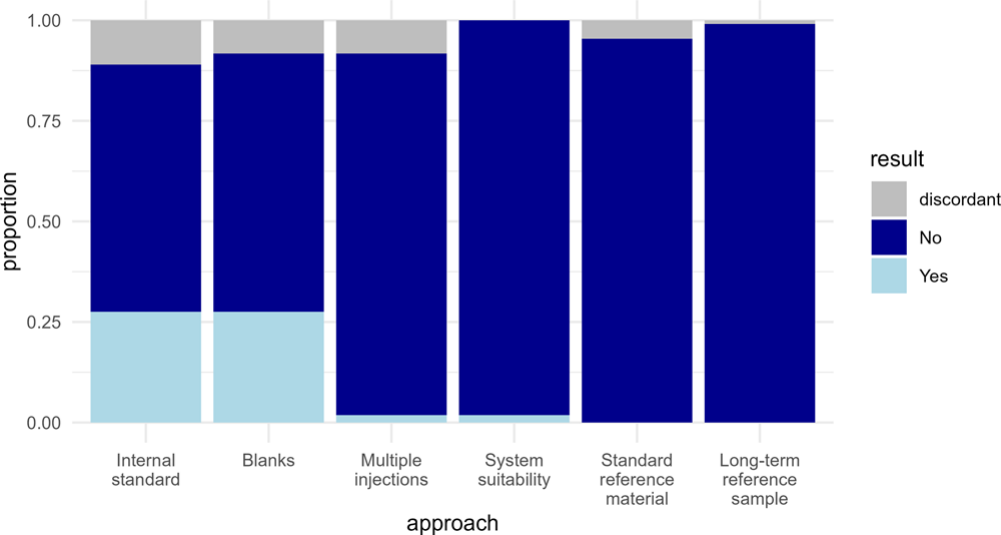
Alternative QC approach frequency. All 109 papers are considered, with results presented independent of pooled QC usage.

Pooled QC samples are one approach to enable objective descriptions of data quality. The current study specifically queried the literature with pooled QC sample approaches in mind but also tallied other approaches that may have been used as part of quality management (Figure 4). These included the possible use of internal standards, blanks, and system suitability samples to assess the overall quality and/or reproducibility for sample preparation, LC-MS measurements, and/or ensuring the system is fit for use prior to LC-MS analysis.

Internal standards and blank samples were the most frequently reported additional QC approaches, each reported in 28% of papers. Furthermore, multiple injections of each study sample or system suitability sample were each reported in 2% of the 109 surveyed papers. Surprisingly, no papers reported the use of standard or long-term reference materials. There was a slight trend for studies that reported the use of pooled QC samples to report the use of internal standards. Of 72 papers which reported use of a pooled QC sample approach, 41 did not report use of an internal standard, 23 reported use of internal standard, and 8 responses were discordant; however, this trend did not reach significance by the χ^2^ test (*p* = 0.484), even after removing all discordant responses (*p* = 0.137). Only six of the 109 surveyed papers cited prior literature reports in describing the QC approach.

### Survey Quality

As described above, each paper was reviewed by at least five reviewers. Answers were determined to have reached “consensus” when at least two-thirds of responses for a single question were identical. In the event that the reviewers did not agree, the answer was assigned as “discordant”. The frequency of discordant answers was very dependent on the paper being reviewed: for the ten most discordant papers, 33% of the responses were discordant, while for the ten most concordant papers, the discordance rate was 3%. The full range of discordance frequency for all papers was from 1 to 41% discordance (see supplemental_5_details.pdf, Figure s3).

Likewise, some questions resulted in more discordance than others (range of 0–46%). However, discordance among reviewers was found to have a much smaller range (12–26%) when compared to the paper or question. To address the question of whether the discordance rate is driven more by paper, question, or reviewer, the variance was tabulated by each factor independently and compared statistically. Equality of variance testing by both *F*-test and Bartlett’s test indicates that the discordance variance is significantly lower by reviewer than paper or question, indicating that the survey respondents are a much smaller source of variance than the paper or the question (Table 1). These data indicate that the review process was robust and suggest that the observed levels of discordance derive primarily from ambiguity in the reported QC methods used.

**Table 1:** Sources of Survey Result Variance

Comparison	Variance ratio	*p* (*F*-test)	*p* (Bartlett’s test)
Reviewer *vs* Paper	0.158	<0.001	<0.001
Reviewer *vs* Question	0.110	<0.001	<0.001
Paper *vs* Question	0.696	0.139	0.229

## Discussion

This literature scoping review analyzes the reported frequency of pooled QC sample usage in LC-MS-based untargeted metabolomics studies. Approximately 32% of relevant literature from the first half of 2021 was surveyed, and the random sampling approach enabled extrapolation of our results to all of the primary research using LC-MS-based metabolomics, providing a high-quality snapshot of pooled QC sample usage for this time period. It is important to note that the language regarding QC samples in the analyzed studies reflects both how QC samples were used and the reporting of that usage. As such, this description may not be a fully accurate representation of what was actually performed in the laboratory. However, the use of multiple reviewers for each paper provides a measure of presentation ambiguity, a valuable metric that specifically reflects reporting.

### Pooled QC Adoption and Use

Approximately two out of every three LC-MS metabolomics papers surveyed reported using a pooled QC sample approach. The pooled QC sample approach was the most widely adopted QC approach in the survey responses, with internal standards and blanks each reported as being used less than half as often as pooled QC samples. Conversely, reporting on how pooled QC samples were used appeared to be much more sporadic. The most frequently reported use of pooled QC samples was to provide an estimate of repeatability. There is no widely accepted metric for delineating acceptable from unacceptable data quality; it is up to individual investigators to determine if their data are reproducible or if corrections are required. Relatively few studies used the pooled QC samples actively to improve data quality (feature filtering, drift correction, and conditioning the LC column). These data collectively suggest that authors and journals recognize the importance of pooled QC sample usage as descriptive of data quality but lack the appropriate software, guidance, or motivation to report the data details of data quality or make more active use of the pooled QC sample approach to improve data quality.^3^

Pooled QC samples are particularly important for identifying technical factors that may impact the observed statistical results. Artifactual trends derived from analytical drift (in signal intensity or mass assignment) over the course of an analytical run, within or among analytical batches, may translate to false positive or false negative statistical results. PCA models can show trends in data, but even relatively large analytical variation can seem insignificant if the biological variation is appreciably larger than the technical variation. It was notable that only one publication used a numerical metric in the PCA analysis to assess the technical variation. PCA is also inherently multivariate, which can be seen as both a strength and a weakness. Standard univariate descriptors (coefficient of variance and linearity in a dilution series) could also be used as descriptors or filters. Only approximately 25% of papers that use pooled QC samples also use feature variance metrics as a means to remove analytically poor features from the data set. There are clear opportunities for the community to increase the use of pooled QC samples in improving data quality, especially considering that the pooled QC samples are frequently being generated and analyzed with the full data set. A recent publication offers guidance on pooled QC usage and reporting,^2^ in which readers can find specific recommendations surrounding pooled QC usage.

Additional QC measures are used in LC-MS metabolomics applications. The data presented here suggest that these alternate approaches, including internal standards, blanks, system suitability samples, replicate injections, long-term reference materials, or standard reference materials, are reported far less frequently than pooled QC samples. Internal standards and blanks were each used in approximately one out of every four papers, and all other approaches were used more infrequently still. Internal standards can be used to enable quantification, permit detection of outliers, and provide a real-time estimate of analytical variance across all study samples, among other uses. Blank samples can provide insight into “contaminant” signals which derive from the extraction process, reagents, or consumables and enable filtering of these contaminants from the data set. These approaches can be considered complementary to the use of a pooled QC sample. However, 17 of the 109 studies reviewed here neither report the use of a pooled QC sample nor clearly report the use of any other QC approaches included in the survey. The lack of reported QC practices potentially reduces confidence in the findings since the reader cannot make an evidence-based assessment of the technical quality of the reported results and the associated conclusions.

### Ambiguity in Reporting

A scientific publication should describe the methods used with sufficient detail to enable replication of the results, though this target is rarely fully met.^21−23^ In practice, the literature reflects some combination of the methods used and the quality of the descriptions of those methods. The literature survey implemented here reveals appreciable ambiguity in descriptions of the manner in which the pooled QC samples were generated; the frequency at which the samples were injected; and the way the pooled QC sample was used in filtering, data correction, and annotation. This ambiguity constrains the accuracy of the current survey-based estimates of QC use frequency but also serves to highlight the importance of accurate and clear reporting. Discordance, a measure of ambiguity derived from multiple reviews of the same paper by several reviewers, arose from either ambiguity in the language describing the pooled QC sample usage in the paper or from the review process itself. The results presented in Table 1 indicate that most of the variance in survey response discordance stems from the question being asked and the language used in the methods for each specific publication, i.e., the author’s description of the QC practice used for that study. As an example of how this discordance can arise, when the answer was not explicitly provided in the paper, one reviewer might have answered the question based on other context provided within the paper, while another may have answered “unclear.”

Some questions were more likely to be classified as discordant than others. The most discordant questions included those surrounding the methods used in generating the pooled QC sample and questions which tended to require more detail than is typically included in a methods section, including the following:

(1) For solid sample matrices, the pooled QC sample was reported to have been created ___. If multiple matrices, answers below assume that the pooled QC is generated from one matrix type only. (choose the best answer.)

- Directly from a solid sample homogenate pre-extraction (i.e. before adding extraction solvent)
- From the sample extract during the extraction process
- From the reconstituted - extracted samples
- Unclear
- Other:

(2) For biofluids (e.g., serum, plasma, urine), the pooled QC sample was reported to have been created ___. (choose the best answer)

- Directly from the biofluid pre-extraction
- From the sample extract during the extraction process
- From the reconstituted extracted samples
- Unclear
- Other:

(3) If pooled QCs were reported to have been used for conditioning the system, how many injections of the pooled QC sample were performed to ensure conditioning of the LC-MS system before beginning an assay? (choose the best answer)

- 1 to 5
- 6 to 10
- 10 or more
- Until certain criteria are met
- Not applicable: pooled QC was not used for conditioning
- Unclear

(4) At which position within the batch were QC samples reported to have been injected ___? (choose all that apply)

- At the beginning of the batch
- In the middle of a batch
- At the end of the batch
- Unclear

(5) Which criterion was reported to have been used with a pooled QC sample to filter features with low precision? (choose the best answer)

- Peak area RSD filter threshold of 10% or less
- Peak area RSD filter threshold of 11 to 20%
- Peak area RSD filter threshold of 21 to 30%
- Peak area RSD filter threshold of 31 to 40%
- Peak area RSD filter threshold of 41% or greater
- Not applicable: Pooled QC samples were not used for this purpose
- Unclear
- Other:

The high rates of discordancy in response to the above questions suggest that authors generally do not prioritize reporting the details describing how the pooled QC sample was generated nor how frequently and in what position the pooled QC sample was injected. The ambiguity in reporting these details changes, for example, how a reader would interpret figures depicting PCA-score plots containing both pooled QC and study samples. Without this knowledge, it is difficult to assess whether pooled QC sample variance derives from LC-MS variance, sample preparation variance, or both. Perhaps unsurprisingly, descriptions of pooled QC sample preparation were more clearly reported for liquid samples than solid. Solid samples generally require additional preparation steps, making it more difficult to concisely describe the preparation of pooled QC solid samples within journal page constrains, though online supplemental methods should alleviate this issue. Importantly, deposition^24,25^ of all raw data coupled with accurate reporting of the location of pooled QC sample injections within the full analytical sequence can enable reprocessing of existing data as algorithms and processing workflows improve.

It is likely that the pooled QC (and alternate QC) sample usage frequencies reported here underrepresent reality; authors may not fully report all the QC methods used, artificially lowering our estimates of use frequency. When QC practices implemented in the laboratory are not reported when the data is delivered (which may be a publication, a confidential report, or a metabolomics data repository^24,25^), an important function of the QC process—to establish and convey confidence in the quality of the reported data—is left unfulfilled. The importance of accurate reporting of quality assurance and QC has been discussed at length recently,^3,4^ and the data reported here clearly support the notion that more accurate reporting is critical.

### Recommendations for Pooled QC Sample Usage and Reporting

The following suggestions are derived from prior literature and the survey results described herein. Please note that these are generalized statements, and the details of implementation will fall upon the scientists involved and may vary between applications.

1. QC procedures should be used to provide objective metrics of data quality, and pooled QC samples are particularly well-suited to QC approaches for untargeted metabolomics.^4,6^
2. The full details describing the preparation and use(s) of any QC samples should be clearly reported in the paper, including criterion used for filtering or acceptance of the data set, if applicable. Guidance for this purpose has been previously described by Kirwan et al.^3^
3. All studies should report the detailed usage on any other quality control approach to ensure the reader is able to assess data quality.
4. While reported use of pooled QC samples is high, this survey suggests that the pooled QC sample could be employed by the community at much higher frequency for improving data quality. Specifically, use of a pooled QC sample in feature filtering (based on either coefficient of variance or dilution series linearity, for example), batch or run order correction, and metabolite identification are infrequently reported and therefore represent a community-wide opportunity to improve data quality.^3^
5. Journals and reviewers should evaluate submitted manuscripts with quality control in mind, and the quality control approach used should be suitable to clearly demonstrate data quality for the reader. Using a template to accurately record this can be useful to ensure all data is accurately recorded.^3^ The reviewers and editors should ensure that
  (a) Technical repeatability/reproducibility of the study was evaluated in an appropriate way, either using pooled QC samples, or other acceptable approaches.
  (b) Pooled QC approach is sufficiently described in methods including its preparation, frequency of injection, evaluation criteria, types of use, etc.
  (c) The results of pooled QC sample evaluation are clearly presented either in the main manuscript or Supporting Information.
  (d) If pooled QC was used to improve data quality, clear description of the approach is provided in the manuscript.
  (e) QC data is deposited together with study data in the data repository, if applicable.
6. QC data should be deposited with the sample data in metabolomics repositories such as MetaboLights^24^ or Metabolomics Workbench.^25^ Doing so will increase transparency and reproducibility while also enabling reprocessing of data with appropriate QC assessment as algorithms and workflows evolve.
7. The community would benefit from an open access and dynamic set of guidance documents describing current best practices. The metabolomics Quality Assurance and Quality Control Consortium (mQACC, https://www.mqacc.org/) has initiated this effort to enable researchers, journal editors, and peer reviewers easy access to current best practice guidelines. This document will contain specific guidance on implementation of pooled QC samples under various scenarios, including for various sample types, study sizes, and analytical platforms. It will also discuss best practices relating to other QC approaches, including use of blanks, internal standards, system suitability testing, and reference materials.

## Conclusions

The literature scoping review described herein indicates that the field of untargeted metabolomics has largely embraced pooled QC samples. However, publication language describing how the pooled QC samples were generated and used is often ambiguous, potentially negatively impacting the confidence in the reported results. Additional QC approaches (internal standards, blanks, etc.) are also in use but are reported at a lower frequency than pooled QC samples, and 17% of articles report no QC whatsoever. The provided recommendations should help guide future efforts to bolster the existing strengths and address the inconsistencies in pooled QC usage and reporting in the metabolomics community.

## Supporting Information Available

Supplemental_1_literature.surveyed.xlsx: a tabular description of the literature surveyed for this study; Supplemental_2_fullSurvey.pdf: the full survey including all questions and answers, as used in this study; Supplemental_3_individual.responses.xlsx: each individual response from each respondent; Supplemental_4_analysisRmarkdown.Rmd: Rmarkdown document used to generate all figures; Supplemental_5_detailed.output.pdf: PDF output from supplemental_4_analysisRmarkdown.Rmd (ZIP)
