# Supplementary material for: Current Practices in LC-MS Untargeted Metabolomics: A Scoping Review on the Use of Pooled Quality Control Samples
Source: Anal Chem. 2023 Dec 6;95(51):18645–54. doi: 10.1021/acs.analchem.3c02924 (PMC10753522; doi:10.1021/acs.analchem.3c02924)
Supplement: Supplementary file 1 — ac3c02924_si_001.zip [file ac3c02924_si_001.zip › supplemental_2_fullSurvey.pdf]

# mQACC pooled QC sample Literature Survey

* Required

1. Last name of reviewer *

2. First name of reviewer *

3. Publication ID (from excel sheet or file name - 3 digits) *

General study information

4. What sample types are reported to have been analyzed in this study (chose all that apply)? *

   Check all that apply.

   - [ ] Mammalian (i.e. biofluids or tissues derived from rat; mouse; human; cat; etc)
   - [ ] Non-mammalian animal (i.e. birds, invertebrates; insects; reptiles; fish...)
   - [ ] Plant (i.e. Arabidopsis; Rice; Algae)
   - [ ] Microbe (i.e. E. coli; Yeast; prokaryote; archeae; cyanobacteria)
   - [ ] Other: ____________

5. How many matrices were analyzed in this study?

Mark only one oval.

- ⬭ 1
- ⬭ 2
- ⬭ 3
- ⬭ 4
- ⬭ More than 4

6. How many independent biological samples per matrix and platform were reported to have been analyzed using untargeted methods? Two tissues from the same sample count as one sample for this classification. Two timepoints from the same sample are classified as two samples. Two individuals will always count as two samples. *

Mark only one oval.

- ⬭ 1 to 50
- ⬭ 51 to 200
- ⬭ 201 to 1000
- ⬭ greater than 1000

7. Was a pooled QC reported to have been used in this study? Note that 'Pooled QC' refers specifically to a quality control sample or samples derived from the experimental samples or extracts. Quality controls such as standard reference material do not qualify as pooled QC samples. *

Mark only one oval.

- ⬭ Yes
- ⬭ No *Skip to question 24*
- ⬭ Unclear *Skip to question 24*
- ⬭ Other: ______________________

Pooled QC Details: (1) your answers should be restricted to only what is described in the paper and its supplemental materials. (2) You should not follow references to other papers which further describe methods . (3) All questions refer only to the untargeted portion of the work. (4) If multiple platforms or matrices were used, please report the maximum QC approach reported.

8. The pooled QC sample was reported to have been generated from equal aliquots of _____. (choose the best answer) *

Mark only one oval.

- ( ) a subset of all biological samples for a given sample matrix
- ( ) all biological samples for a given sample matrix
- ( ) all samples for a given sample matrix - including process blanks
- ( ) all samples, including multiple matrices
- ( ) Unclear
- ( ) Other: ______________________

9. Was metabolomics performed on solid samples? (tissues, dried blood spots, etc)

Mark only one oval.

- ( ) Yes *Skip to question 10*
- ( ) No *Skip to question 11*
- ( ) Unclear

Solid sample pooled QC section

10. For solid sample matrices, the pooled QC sample was reported to have been created ___________. If multiple matrices, answers below assume that the pooled QC is generated from one matrix type only. (choose the best answer.) *

Mark only one oval.

- Directly from a solid sample homogenate pre-extraction (i.e. before adding extraction solvent)
- From the sample extract during the extraction process
- From the reconstituted - extracted samples
- Unclear
- Other: ____________________

Liquid sample pooled QC section

11. Was metabolomics performed on liquid samples? (plasma, urine, surface water, etc)

Mark only one oval.

- Yes *Skip to question 12*
- No *Skip to question 13*
- Unclear *Skip to question 13*

Liquid sample pooled QC section - details

12. For biofluids (e.g., serum, plasma, urine), the pooled QC sample was reported to have been created _______. (choose the best answer) *

Mark only one oval.

- ⬭ Directly from the biofluid pre-extraction
- ⬭ From the sample extract during the extraction process
- ⬭ From the reconstituted extracted samples
- ⬭ Unclear
- ⬭ Other: ______________________

Pooled QC Usage

13. How was the pooled QC sample reported to have been used? (choose all that apply) *

Check all that apply.

- [ ] Conditioning the LC-MS system
- [ ] Providing an estimate of reproducibility
- [ ] Batch or Drift correction
- [ ] Filtering features during data processing
- [ ] Metabolite identification
- [ ] Unclear or observational only
- [ ] No reported use of pooled QC
- [ ] Other

14. If pooled QCs were reported to have been used for conditioning the system, how many injections of the pooled QC sample were performed to ensure conditioning of the LC-MS system before beginning an assay? (choose the best answer) *

Mark only one oval.

- 1 to 5
- 6 to 10
- 10 or more
- until certain criteria are met
- Not applicable: pooled QC was not used for conditioning
- Unclear

15. Pooled QC samples were reported to have been injected ________. (choose the best answer) *

Mark only one oval.

- One time *Skip to question 24*
- Multiple times *Skip to question 16*
- Unclear *Skip to question 24*

Multiple injections of QC samples

16. Multiple QC injections were reported to have been made from ____.

Mark only one oval.

- One vial
- More than one vial
- Unclear

17. At which position within the batch were QC samples reported to have been injected _____________? (choose all that apply) *

Check all that apply.

- [ ] At the beginning of the batch
- [ ] In the middle of a batch
- [ ] At the end of the batch
- [ ] Unclear

18. At what frequency were QC samples reported to have been injected?

Mark only one oval.

- ( ) Every 2 to 5 samples
- ( ) Every 6 to 10 samples
- ( ) less frequently than every 10 samples
- ( ) Unclear

19. Was a dilution series of the pooled QC sample reported to have been used? (choose the best answer) *

Mark only one oval.

- ( ) Yes - with 3 or fewer dilution levels
- ( ) Yes - with 4-6 dilution levels
- ( ) Yes - with 7-10 dilution levels
- ( ) Yes - with more than 10 dilution levels
- ( ) No
- ( ) Unclear
- ( ) Other: ______________________

20. Which criterion was reported to have been used with a pooled QC sample to filter features with low precision? (choose the best answer) *

Mark only one oval.

- Peak area RSD filter threshold of 10% or less
- Peak area RSD filter threshold of 11 to 20%
- Peak area RSD filter threshold of 21 to 30%
- Peak area RSD filter threshold of 31 to 40%
- Peak area RSD filter threshold of 41% or greater
- Not applicable: Pooled QC samples were not used for this purpose
- Unclear
- Other: ____________________

PCA usage

21. Were pooled QC samples used in PCA analysis?

Mark only one oval.

- Yes
- No *Skip to question 24*
- Unclear *Skip to question 24*

PCA usage - Details

22. If PCA was reported to have been used to evaluate pooled QC clustering/variability in a study, the analysis included _____. (choose the best answer) *

Mark only one oval.

- all biological samples (for a given sample type) and pooled QC samples
- pooled QC samples only
- Not Applicable: PCA was not used in this study to evaluate pooled QC samples *Skip to question 24*
- Unclear
- Other

23. What criteria were reported to have been used to evaluate pooled QC clustering/variability in the PCA plot? (choose the best answer) *

Mark only one oval.

- Visual inspection
- Quantitative metric
- Unclear
- Other: ____________________

Final Thoughts

24. Was the same QC approach used for all matrices

Mark only one oval.

- Yes
- No
- Unclear
- NA

25. Which - if any - alternate QC samples/approaches were reported to have been utilized in this study? (Choose all that apply) *

Check all that apply.

- [ ] Standard Reference Material or Interlaboratory QC sample
- [ ] Long Term Reference QC sample or Intralaboratory QC sample
- [ ] System suitability sample (i.e. pubmed pid 29805336)
- [ ] Blanks (system; extraction; solvent; etc)
- [ ] Internal Standards
- [ ] Multiple injections of each experimental sample
- [ ] No other QC sample types were used
- [ ] Unclear
- [ ] Other

26. Did this manuscript use citations to previous studies in describing their quality control methods? Answer 'Yes' if the reference suggests there are more details on quality control methods in the referenced paper and 'No' if citations were not used or if the reference suggests it is not related to quality control approach. *

Mark only one oval.

- ( ) Yes
- ( ) No

27. The untargeted work was reported to have been performed at ______. (choose all that apply)

Check all that apply.

- [ ] Independent invesitigator laboratory
- [ ] Core metabolomics facility
- [ ] Commercial metabolomics provider
- [ ] Unclear
- [ ] Other: ____________________

28. Please provide any comments on this review which were not captured in the responses above.

Thank you for input - please 'submit' to ensure your survey responses are logged.

This content is neither created nor endorsed by Google.

Google Forms
