# Supplementary material for: Current Practices in LC-MS Untargeted Metabolomics: A Scoping Review on the Use of Pooled Quality Control Samples
Source: Anal Chem. 2023 Dec 6;95(51):18645–54. doi: 10.1021/acs.analchem.3c02924 (PMC10753522; doi:10.1021/acs.analchem.3c02924)
Supplement: Supplementary file 1 — ac3c02924_si_001.zip [file ac3c02924_si_001.zip › supplemental_5_Details.pdf]

mqacc.pooledqc.lit.survey: detailed summary report

Corey Broeckling, Metabolomics Quality Assurance and Quality Control Consortium

2023-01-12

Table of Contents

Introduction and Approach: 1
Pooled QC Survey Response Summary: 2
Results: Survey Quality and General Study Design Overview 2
Results: Pooled QC Specific Questions 8
Results: For those papers which used a pooled QC approach 12
Results: Other QC Approaches 41
Publication Figures: 54

Introduction and Approach:

This document provides a complete view of the minimally processed and filtered mQACC pooled quality control literature survey. The survey responses were exported as a csv file from google forms/sheets, minimally edited to remove duplicate posts (two responses from the same reviewer of the same paper - the most recent review was kept) and to replace custom sample type descriptions with original categorical descriptions when appropriate (i.e. 'propolis' became 'Plant (i.e. Arabidopsis; Rice; Algae)', 'human cell lines' became 'Mammalian (i.e. biofluids or tissues derived from rat; mouse; human; cat; etc)'). Paper 109 was removed from the survey results as it was found to have been missed in the filter for 'untargeted acquisition' - it only used targeted methods.

To streamline the presentation, we assign a consensus answer to each question for each paper. 'Concordance' is defined as the situation where at least two out of three people agreed. Discordant represents less than 2/3, or roughly < 0.667, keeping in mind that 'nr' answers are removed from the consensus process as described below 'Discordant' therefore can be interpreted as a lack of consensus on a given paper/question.

The survey was structured such that some questions were only conditionally visible - if a reviewer concluded that it was unclear whether a pooled-QC approach was used, the question asking whether the pooled QC was used in PCA was not asked, for example. In these cases, the answer that is coded in this setting is 'nr' for 'no response.' All missing values, whether due to a reviewer forgetting to answer a question or the question not being visible due to the conditional nature of the survey, are coded as 'nr' and treated the same. In assigning the consensus answer, nr was assigned as the consensus only of there was zero or one non-nr answer, i.e. :

- nr, nr, nr, nr, yes would be assigned a consensus answer of ‘nr’
- nr, nr, nr, yes, yes would be assigned a consensus answer of ‘yes’
- nr, nr, nr, yes, no would be assigned a consensus answer of ‘discordant’

Note also that some questions have an ‘Unclear’ answer. ‘Unclear’ always represents a deliberate choice made by one reviewer to one question, and is *not* assigned as part of the ‘consensus’ answer process. Unclear is assigned as the consensus answer only when the majority of people call the answer ‘Unclear’. Thus, in some ways, ‘unclear’ and ‘discordant’ represent two different ways of reporting the absence of a clear consensus answer.

All questions which had ‘choose all that apply’ structure have each option reported individually. ‘Other’ answers were aggregated back into ‘Other’ when appropriate.

For this summary we are not displaying any ‘nr’ values in the summary plots to simplify presentation. The source data can be found in the file ‘supplementary_3_individual.responses.csv’. All reviewers have been made anonymous as integer identifiers.

Pooled QC Survey Response Summary:

Results: Survey Quality and General Study Design Overview

Figure s1. Number of reviews for each paper:

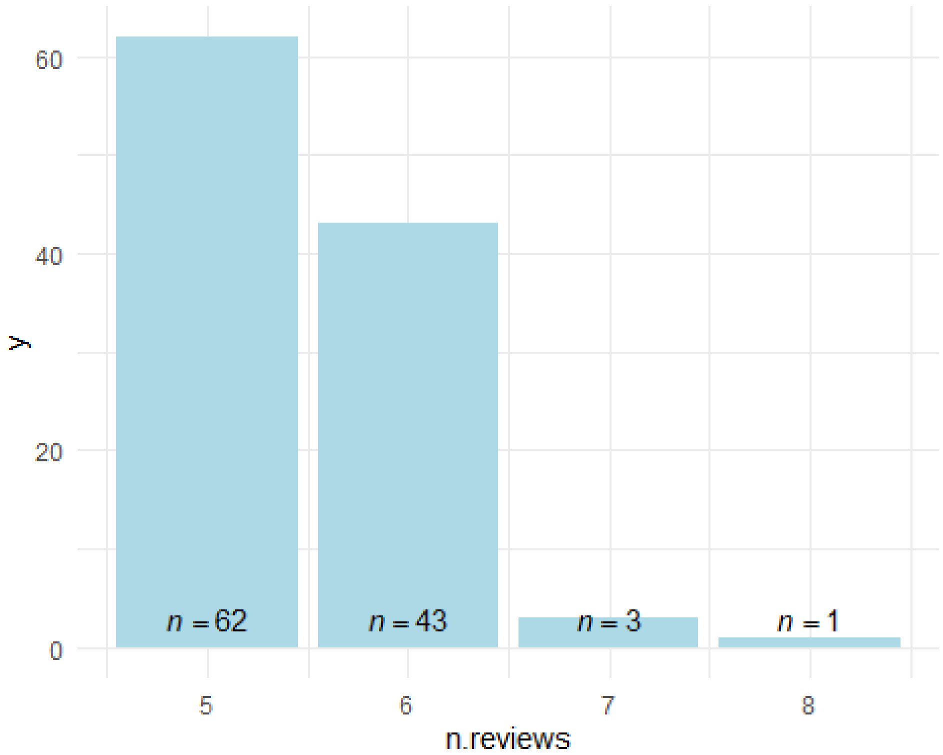

Figure s2. Question 4: What sample types are reported to have been analyzed in this study (chose all that apply)?

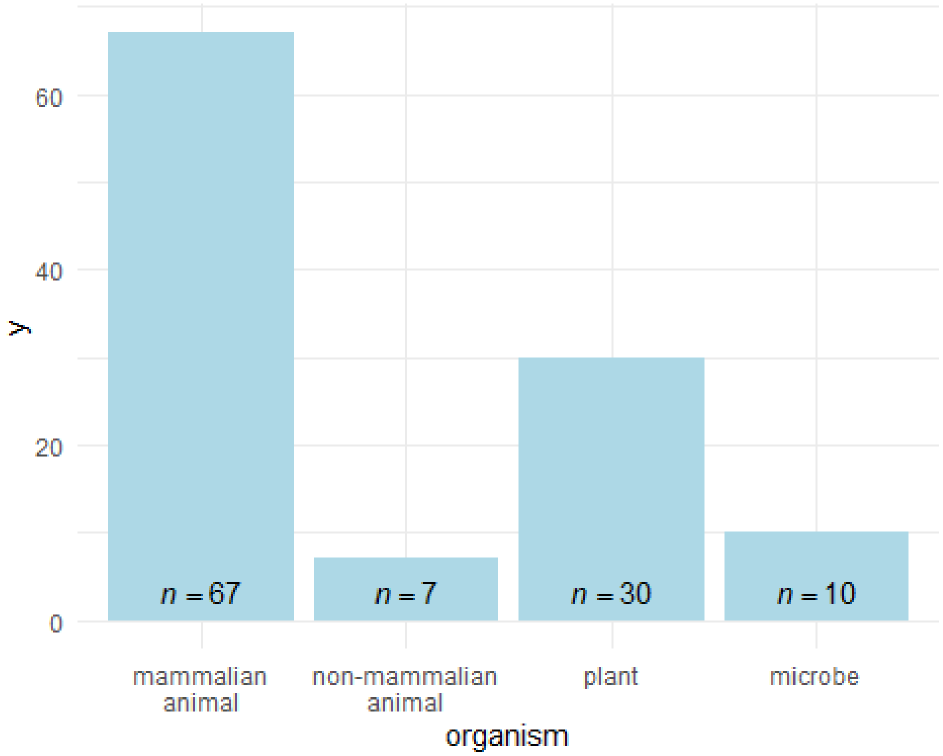

Discordance rate by paper:

Every individual paper has a percentage of question responses which are discordant (no consensus was reached). This percentage could range from zero to one hundred percent. Some papers have high percentage of questions which are discordant, some are low. Below is plotted a frequency histogram to show the distribution of percent-discordance for all papers. The x-axis is the percentage of answers for a given *paper* which were found to be discordant. The y-axis represents the frequency of those percentage values across all papers. i.e. there are 5 papers with a discordance percentage of approximately 10%. This plot shows that there are many papers which scored a discordance rate of 12% or less. Conversely there are several papers which have a high discordance rate (25% or more).

Figure s3. Histogram of discordance rate by paper:

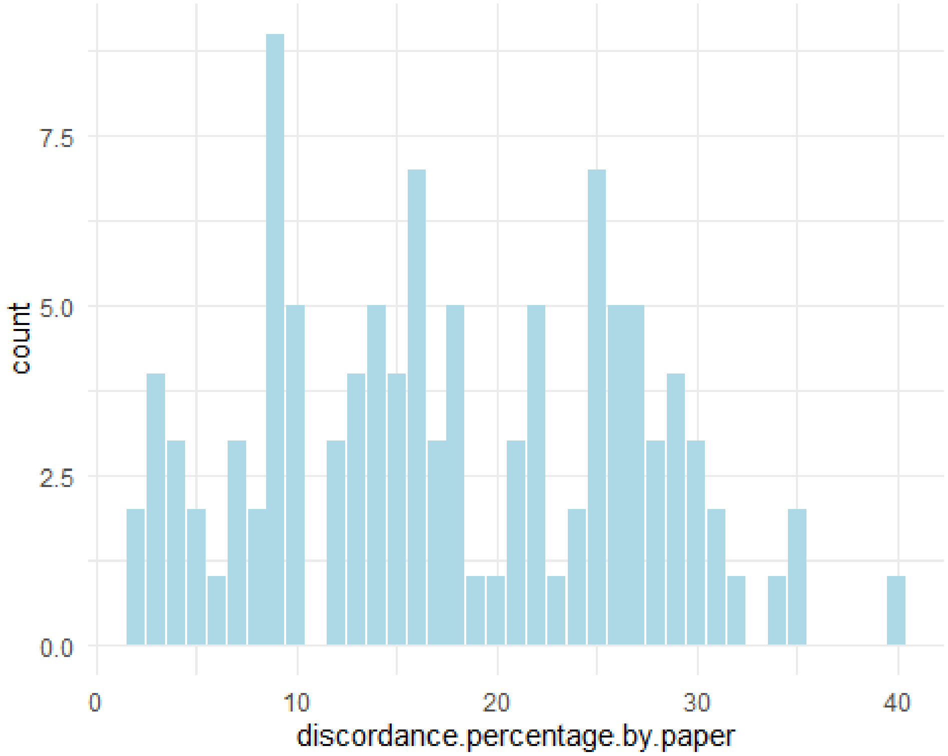

The papers with the most discordant responses include:

- 30, 42, 47, 52, 63, 96, 100, 105, 108, 142, 157, 163, 207

The papers with the most concordant responses include:

- 1, 6, 27, 40, 48, 64, 73, 82, 88, 89, 124, 128, 171, 178, 180, 199, 208, 216, 233

Of the papers listed above:

The discordance rate is defined simply as the percentage of all answers which are flagged as being discordant (not identical to the concordant answer).

- 10 least discordant papers: discordance rate = 33 %
- 10 most discordant papers: discordance rate = 3 %

Figure s4. Histogram of discordance rate by question:

As above, but plotted by question, rather than by paper.

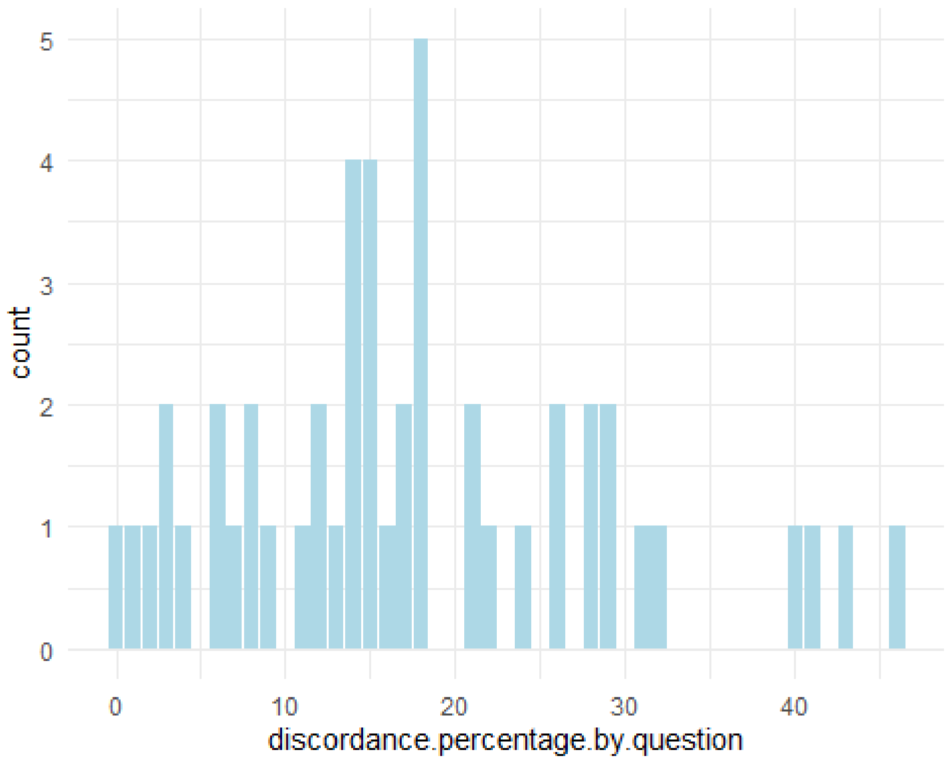

The questions with the most discordant responses include:

- For solid sample matrices, the pooled QC sample was reported to have been created __________. If multiple matrices, answers below assume that the pooled QC is generated from one matrix type only. (choose the best answer.)
- For biofluids (e.g., serum, plasma, urine), the pooled QC sample was reported to have been created ______. (choose the best answer)
- If pooled QCs were reported to have been used for conditioning the system, how many injections of the pooled QC sample were performed to ensure conditioning of the LC-MS system before beginning an assay? (choose the best answer)
- At which position within the batch were QC samples reported to have been injected ___________? (choose all that apply) At the end of the batch
- At which position within the batch were QC samples reported to have been injected ___________? (choose all that apply) Unclear
- Which criterion was reported to have been used with a pooled QC sample to filter features with low precision? (choose the best answer)
- The untargeted work was reported to have been performed at ____. (choose all that apply) Independent invesitigator laboratory

Figure s5. Histogram of discordance rate by reviewer:

As above, but plotted by reviewer, rather than by paper or question.

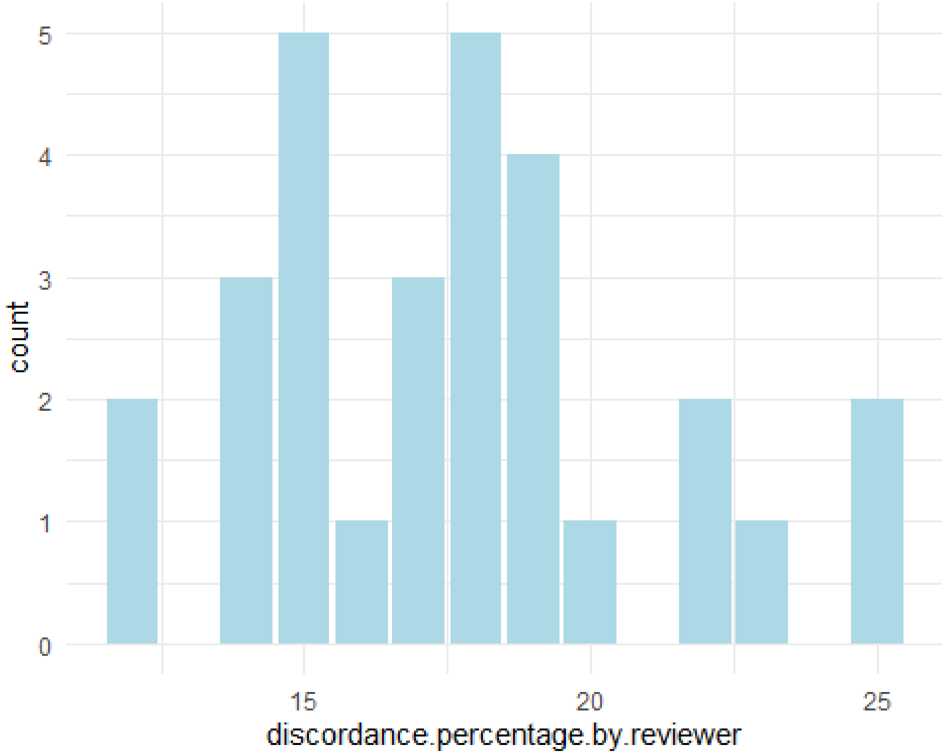

This visualization shows us that the average levels of discordance are (necessarily) similar if we slice by either reviewer, paper, or question. However, the distribution and standard deviation across these three factors is quite different. The table below describes the discordance percentage mean and standard deviation for each factor, as well as the p-value for a the shapiro test for normality of the distribution.

Table s1: Discordant response frequency summary by reviewer, paper, and question.

	source	mean	sd	normality.test.p
1	reviewer	17.62069	3.437335	0.17942420
2	paper	17.74312	9.019941	0.01436238
3	question	17.66667	11.183828	0.04082691

These data indicate that the the distribution for paper and question do not fit a gaussian distribution, while the distribution by reviewer does. Further, the standard deviation values indicate that the variation in discordance is very dependent on the question, less so on the paper, and least of all on the reviewer. These data suggest that the major sources of discordance are paper and question, not reviewer, indicating that the quality of the review is sound, but there are simply some questions and some papers which are inherently more difficult to generate clear consensus answers.

An F-test can be used to compare variance, though the questionable normality (see above p-values) of the percentage distribution by paper and question suggest it may not be the

most appropriate test. A Bartlett test is less sensitive to normality - p values for both are included below. A lower variance ratio indicates lower variance from 'reviewer' than from the comparitive factor. To ensure the sample size of each factor were the same for the Bartlett test, the longer variable was randomly sampled to the same n as the shorter variable (n = 29 reviewers, n = 109 papers, n = 46 questions)

Table s2: Statistical analysis of discordant response frequency summary by reviewer, paper, and question.

```
                    test variance.ratio F.test.p.value bartletts.p.value
1     reviewer.vs.paper     0.14522331   2.178830e-07      3.055206e-06
2  reviewer.vs.question     0.09446321   4.561348e-09      1.058541e-09
3     paper.vs.question     0.65046865   7.018244e-02      1.192055e-01
```

Figure s6. Question 5: How many matrices were analyzed in this study?

```
< table of extent 0 x 0 x 109 x 7 x 4 x 4 x 3 x 5 x 4 x 6 x 4 x 6 x 3 x 3 x 3
x 3 x 2 x 3 x 2 x 3 x 3 x 7 x 4 x 4 x 3 x 3 x 3 x 3 x 3 x 6 x 4 x 7 x 4 x 4 x
4 x 5 x 3 x 2 x 3 x 3 x 2 x 2 x 3 x 3 x 2 x 3 x 3 x 3 x 3 x 3 x 3 >
```

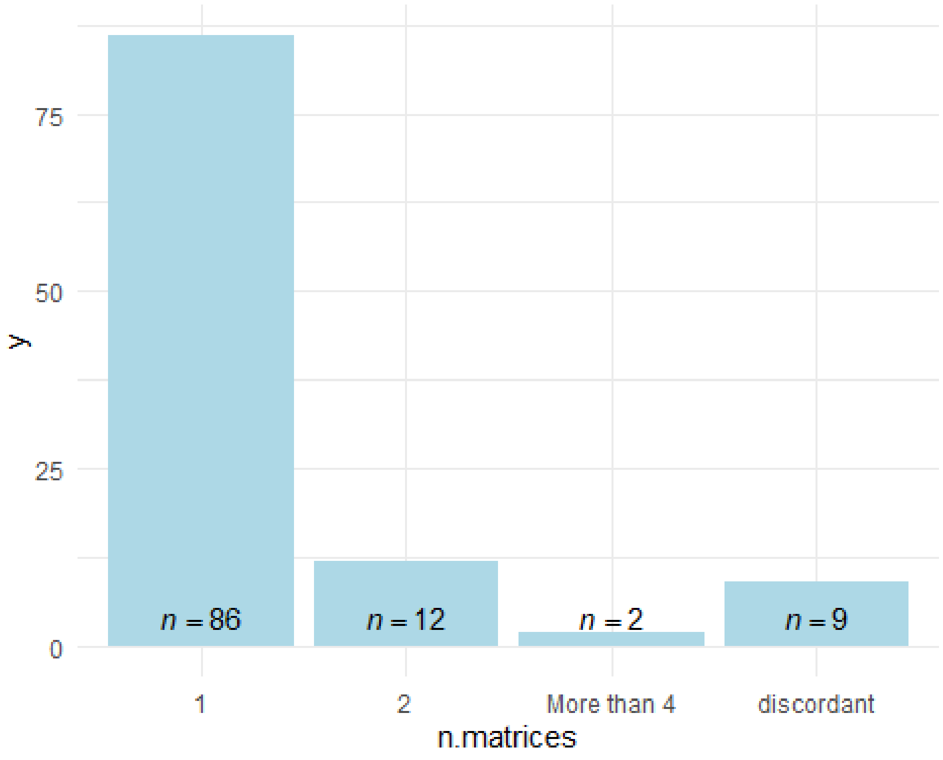

Figure s7. Question 6: How many independent biological samples per matrix and platform were reported to have been analyzed using untargeted methods? Two tissues from the same sample count as one sample for this classification. Two timepoints from the same sample are classified as two samples. Two individuals will always count as two samples.

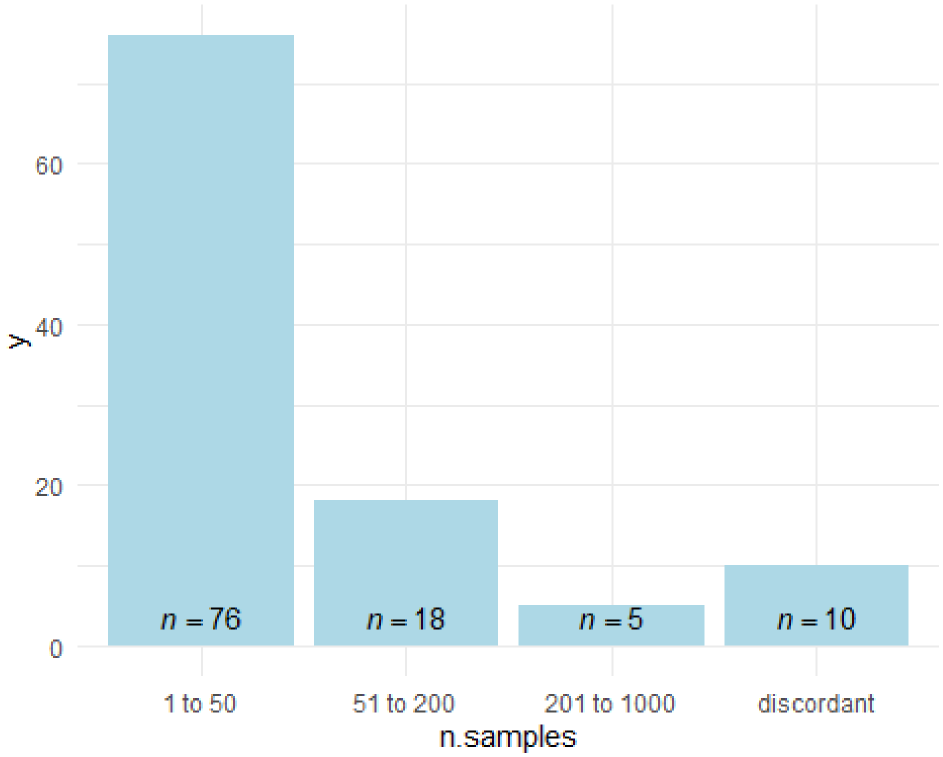

Results: Pooled QC Specific Questions

All the questions below more directly address the study aims: was a pooled QC used and if so how?

Figure s8. Question 7: Was a pooled QC reported to have been used in this study? Note that 'Pooled QC' refers specifically to a quality control sample or samples derived from the experimental samples or extracts. Quality controls such as standard reference material do not qualify as pooled QC samples.

This is the main question of the survey.

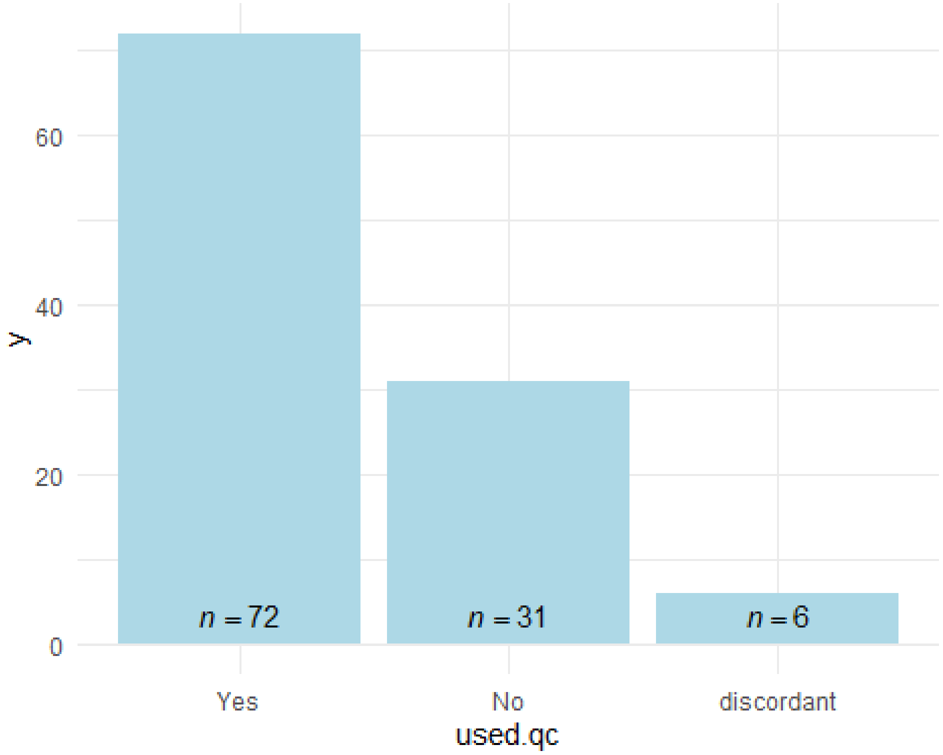

Descriptions of the results from papers which have reported discordance

```
Paper 20
 -  No

 -  Yes

 -  Unclear

 -  No

 -  Yes

 -  Yes
Paper 28
 -  Unclear

 -  other

 -  No

 -  Yes

 -  Unclear
Paper 71
 -  No
```

```
 -  Yes

 -  No

 -  Yes

 -  No
Paper 87
 -  No

 -  No

 -  Yes

 -  Yes

 -  No

 -  Yes

 -  Yes
Paper 167
 -  other

 -  No

 -  Unclear

 -  Unclear

 -  Unclear
Paper 215
 -  No

 -  Unclear

 -  Unclear

 -  Unclear

 -  No

 -  No
```

Figure s9. qc.usage.by.organism

Studies were classified by whether they analyzed samples from mammals, non-mammalian animals, plants, or microbes. The plot below shows the frequency of pooled QC usage by organism class. Note that a given study may be listed more than once if it analyzed more than one organism class.

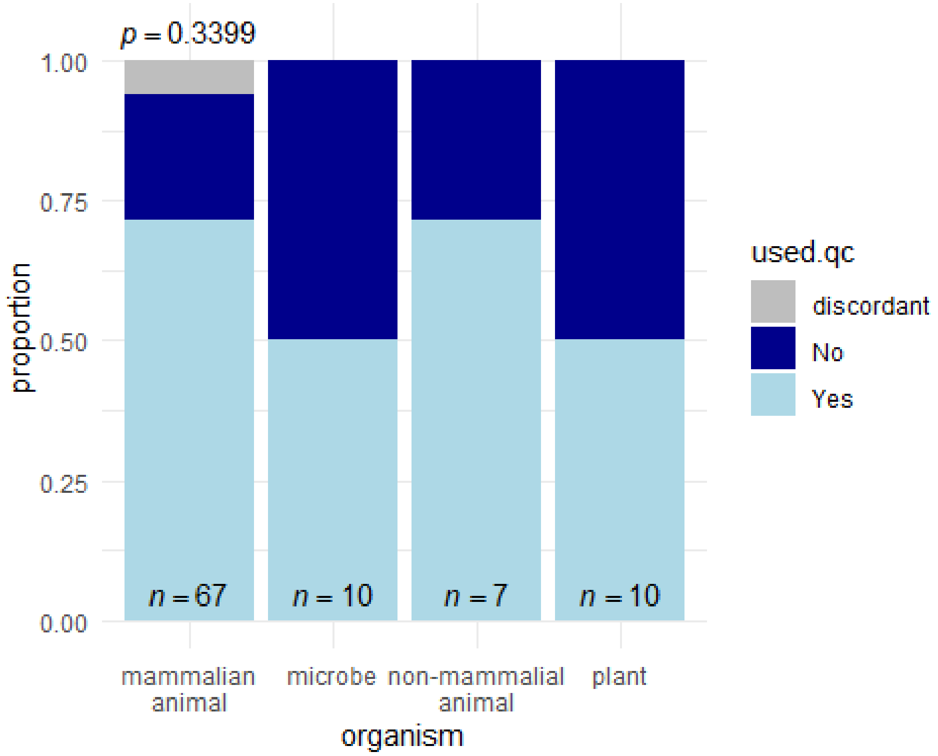

Figure s10. qc.usage.by.sample.size

One might expect that larger studies would be more likely to use a pooled QC approach than smaller studies. This is in fact what is observed, though the trend is not significant by chi-square testing.

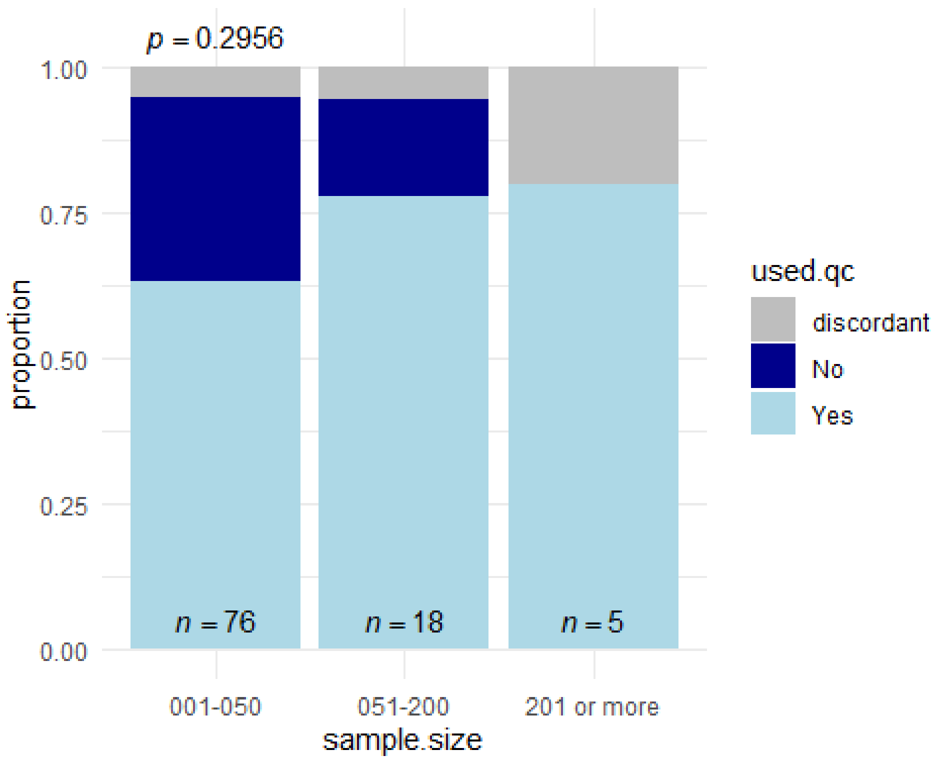

Results: For those papers which used a pooled QC approach

For upcoming questions, a filter was applied such that we are only looking at results for which there was a clear consensus 'Yes' answer to the question 'was a pooled QC used?'. In all plots below the total number of papers being considered is listed in the title to reflect that we are no longer looking at the full 109 paper set, rather only the papers where a pooled QC samples is being used.

Figure s11. Question 8: The pooled QC sample was reported to have been generated from equal aliquots of _____. (choose the best answer)

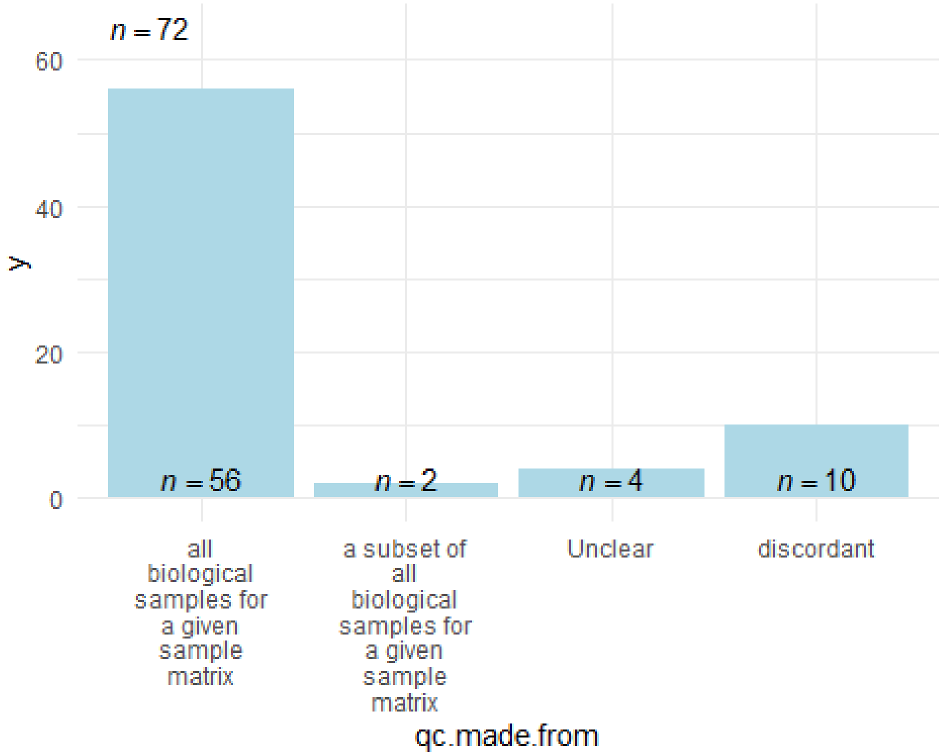

Figure s12. Question 9: Was metabolomics performed on solid samples? (tissues, dried blood spots, etc)

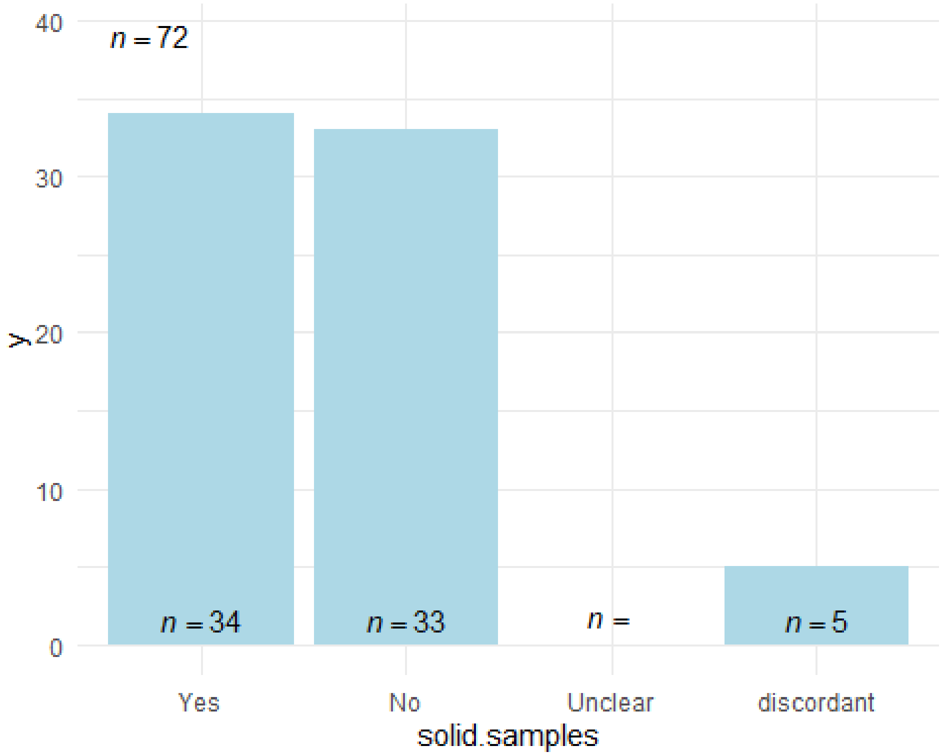

for those papers for which a solid sample type was reported to have been used

Figure s13. Question 10: For solid sample matrices, the pooled QC sample was reported to have been created ____________. If multiple matrices, answers below assume that the pooled QC is generated from one matrix type only. (choose the best answer.)

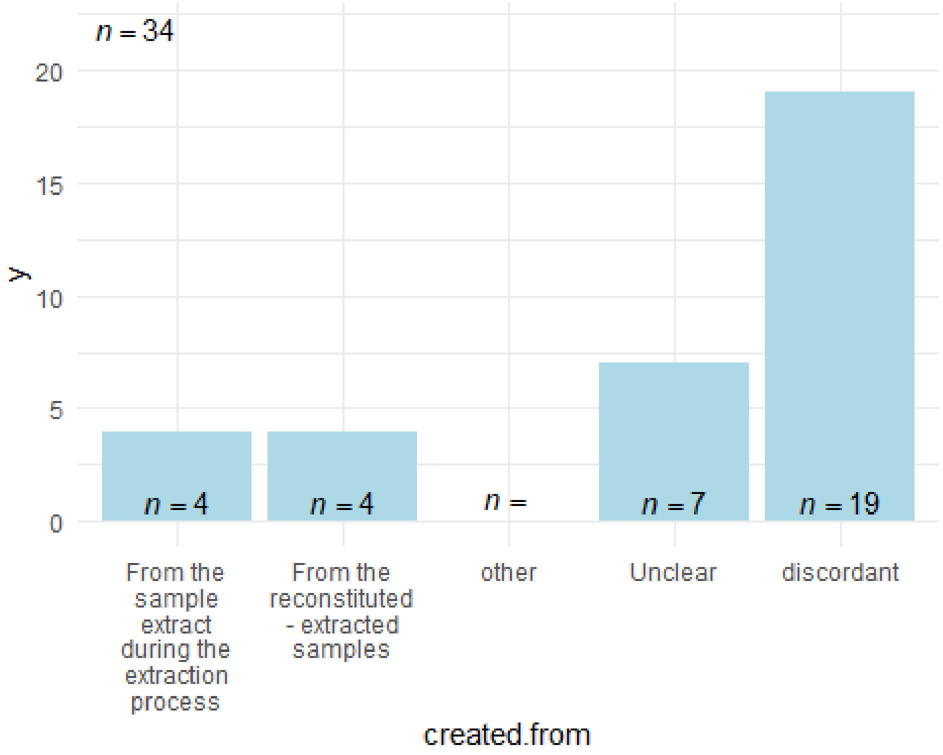

Figure s14. Question 11: Was metabolomics performed on liquid samples? (plasma, urine, surface water, etc)

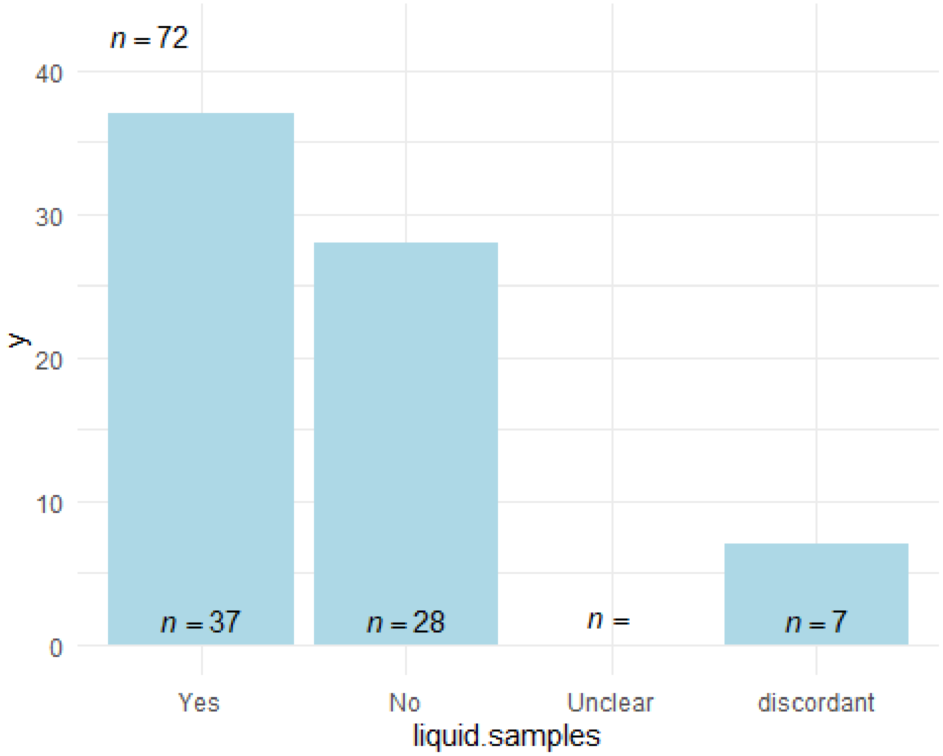

descriptions of the results from papers which have reported discordance

```
Paper 42
 -  Yes

 -  No

 -  No

 -  Yes

 -  No
Paper 63
 -  No

 -  No

 -  Unclear

 -  Yes

 -  Yes
Paper 65
 -  No
```

```
 -  No

 -  Yes

 -  Yes

 -  Yes
Paper 71
 -  nr

 -  No

 -  nr

 -  Yes

 -  nr
Paper 89
 -  Unclear

 -  No

 -  No

 -  Yes

 -  Yes

 -  No
Paper 108
 -  No

 -  No

 -  Yes

 -  Yes

 -  No
Paper 125
 -  nr

 -  nr

 -  nr

 -  No
```

```
 -  nr

 -  Yes
Paper 142
 -  Yes

 -  No

 -  Yes

 -  No

 -  No
Paper 204
 -  Yes

 -  No

 -  No

 -  Yes

 -  No
```

For those papers for which a liquid sample type was reported to have been used:

Figure s15. Question 12: For biofluids (e.g., serum, plasma, urine), the pooled QC sample was reported to have been created ________. (choose the best answer)

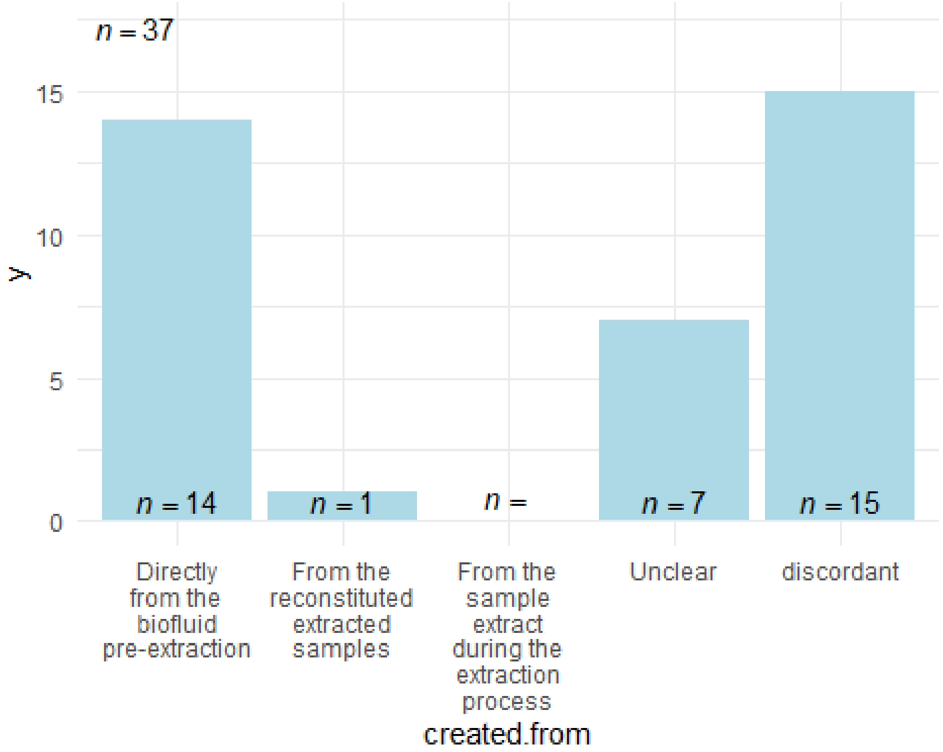

Figure s16. Question 13: How was the pooled QC sample reported to have been used? (choose all that apply) Batch or Drift correction

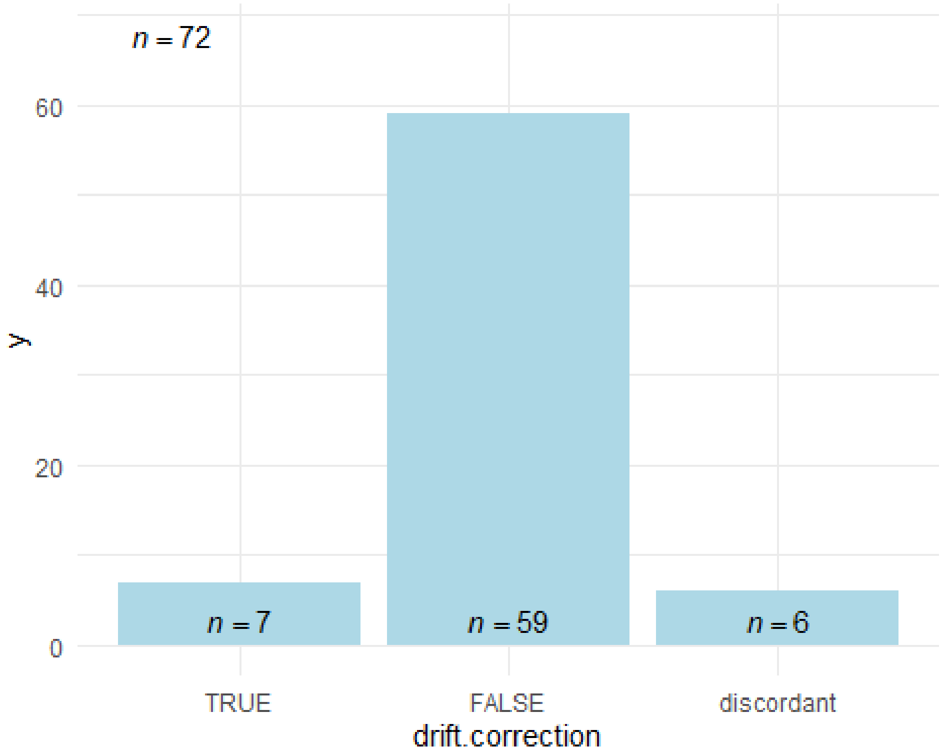

Figure s17. Question 14: How was the pooled QC sample reported to have been used? (choose all that apply) Conditioning the LC-MS system

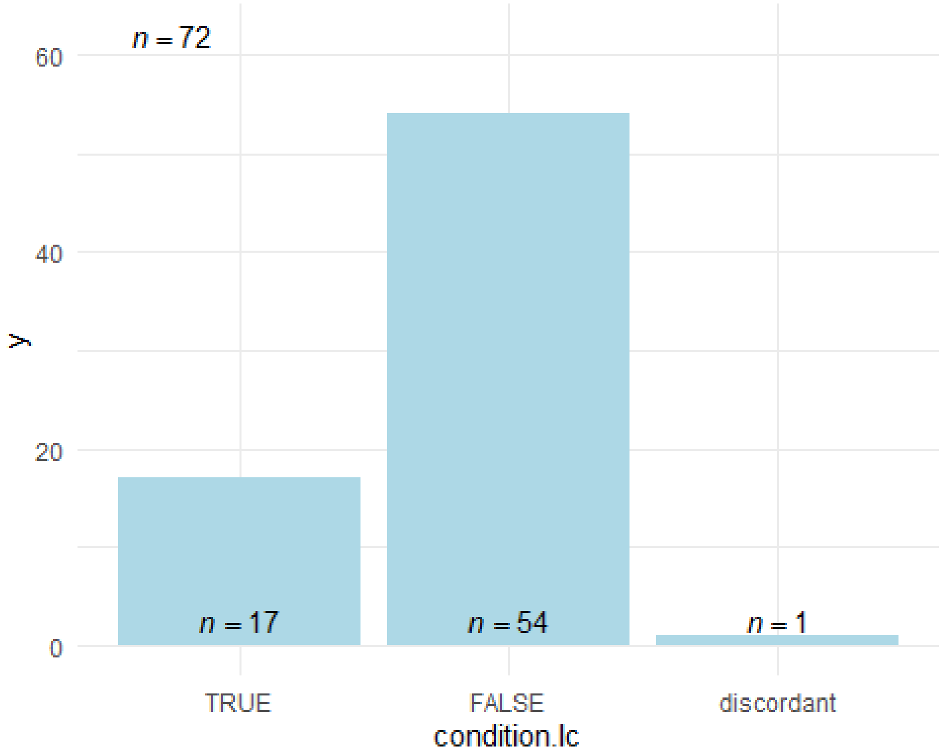

Figure s18. Question 15: How was the pooled QC sample reported to have been used? (choose all that apply) Filtering features during data processing

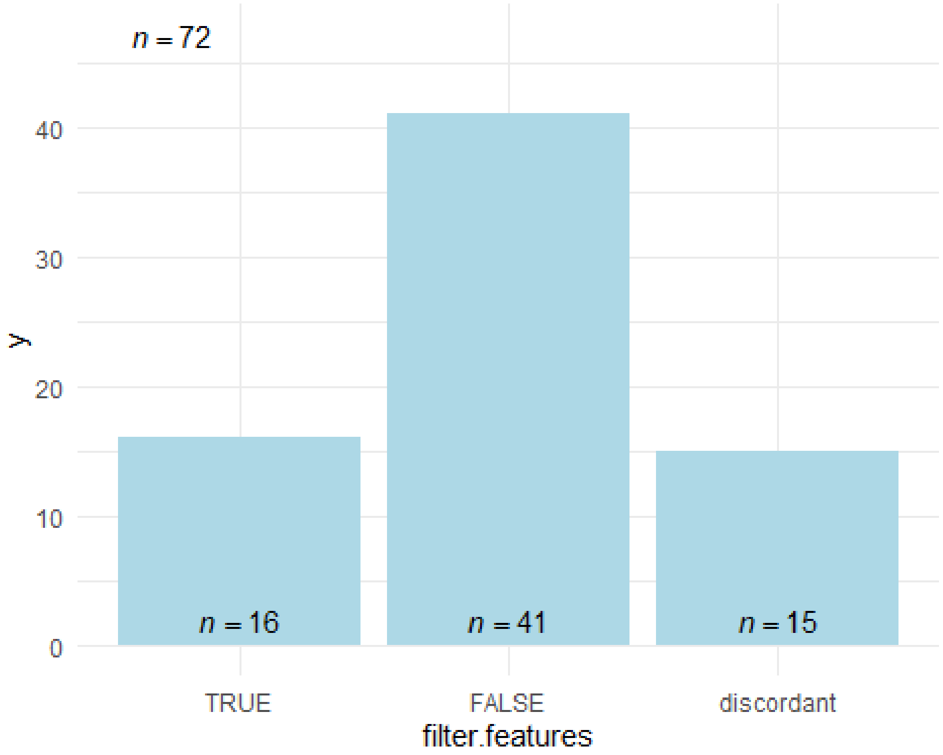

Figure s19. Question 16: How was the pooled QC sample reported to have been used? (choose all that apply) Metabolite identification

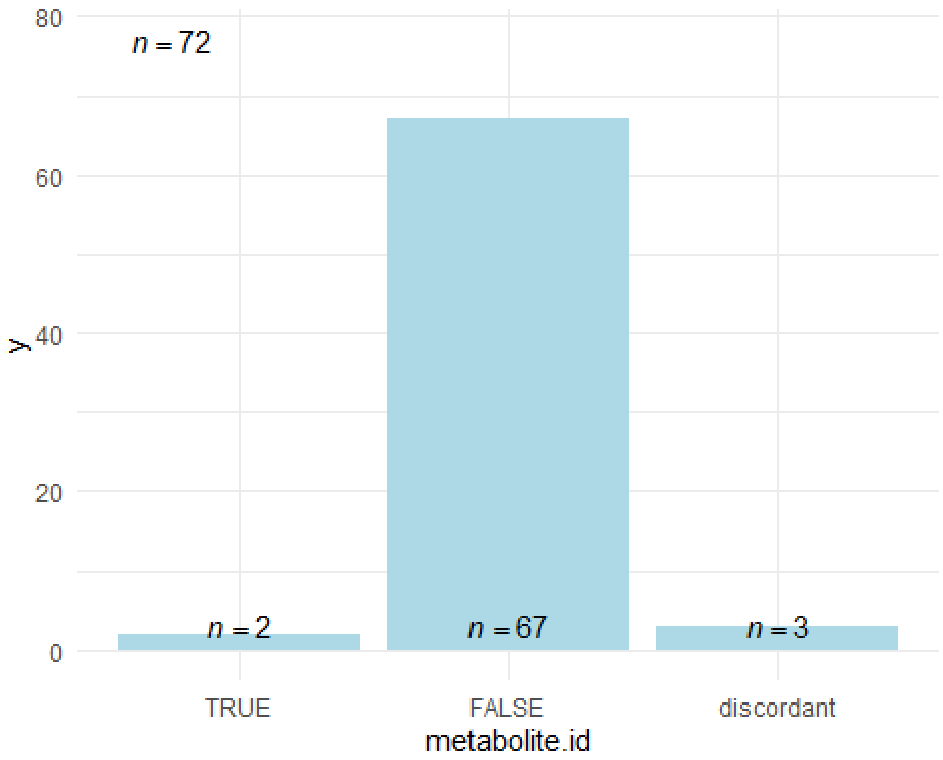

Figure s20. Question 17: How was the pooled QC sample reported to have been used? (choose all that apply) No reported use of pooled QC

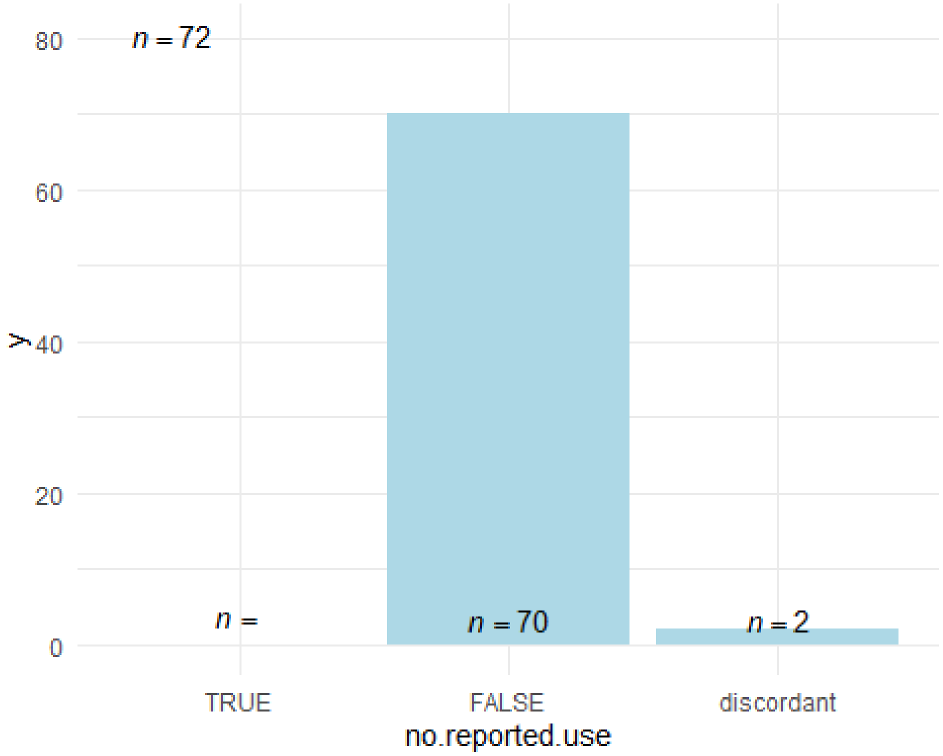

Figure s21. Question 19: How was the pooled QC sample reported to have been used? (choose all that apply) Other

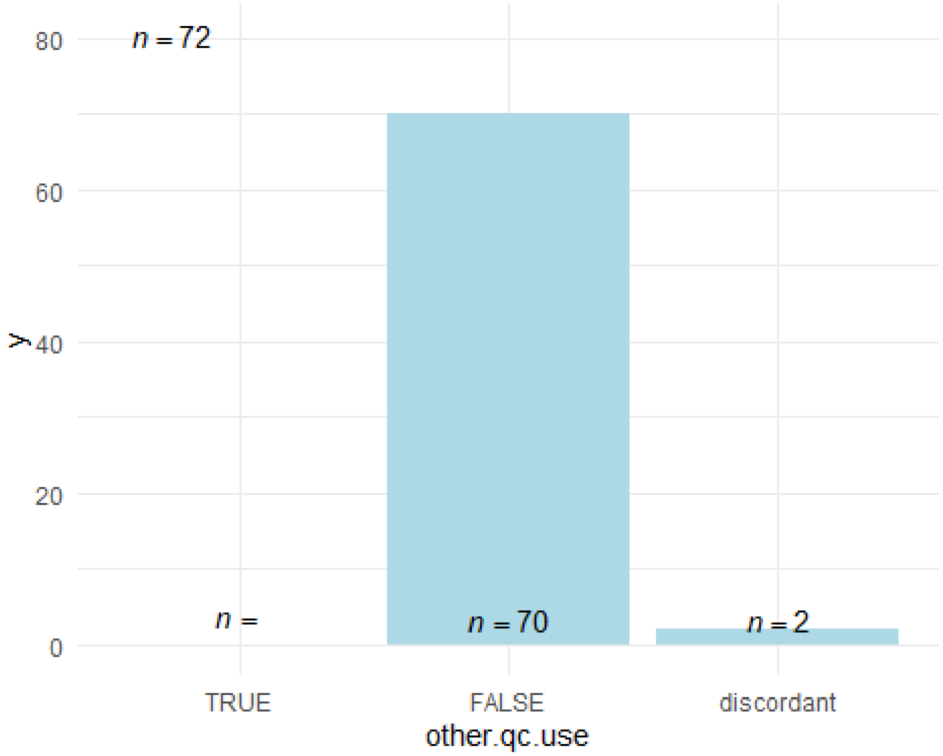

Figure s22. Question 20: How was the pooled QC sample reported to have been used? (choose all that apply) Providing an estimate of reproducibility

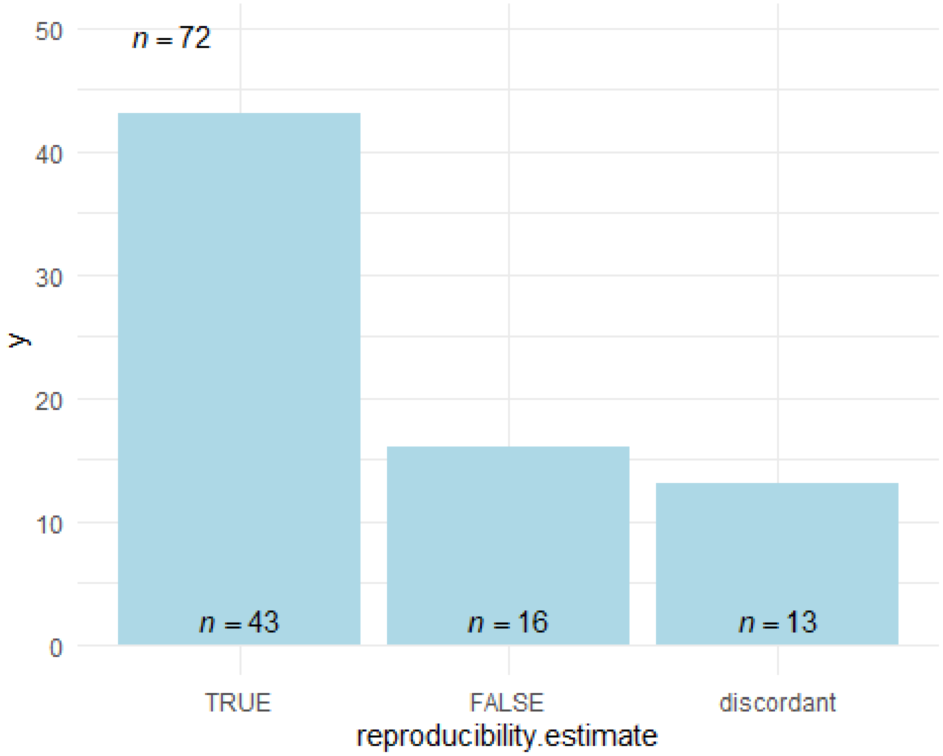

Figure s23. Question 21: How was the pooled QC sample reported to have been used? (choose all that apply) Unclear or observational only

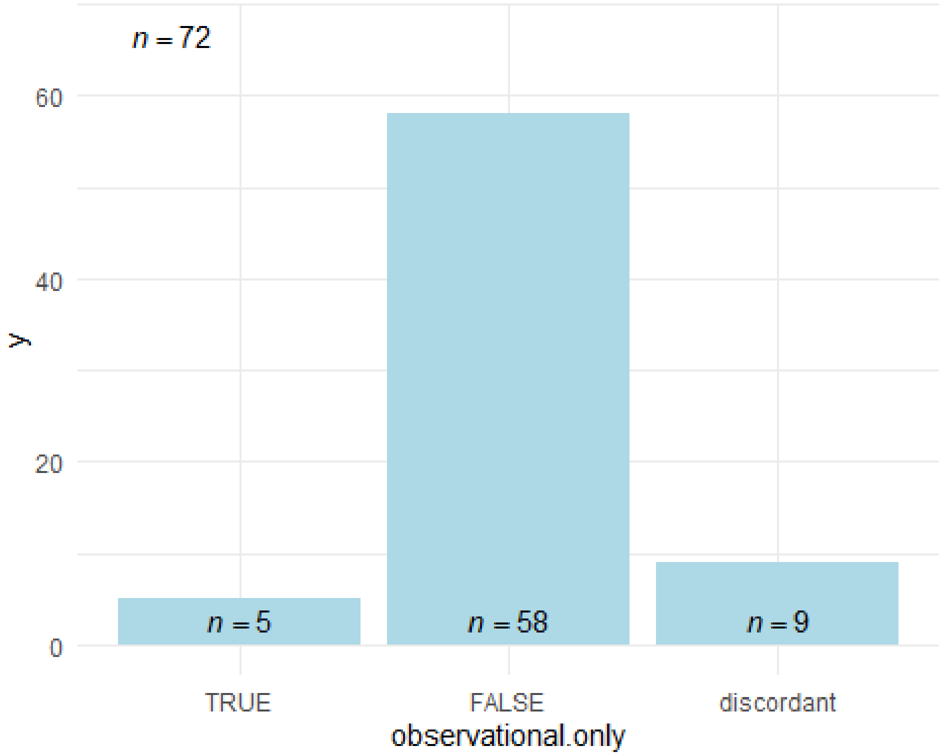

Figure s24. Question 22: If pooled QCs were reported to have been used for conditioning the system, how many injections of the pooled QC sample were performed to ensure conditioning of the LC-MS system before beginning an assay? (choose the best answer)

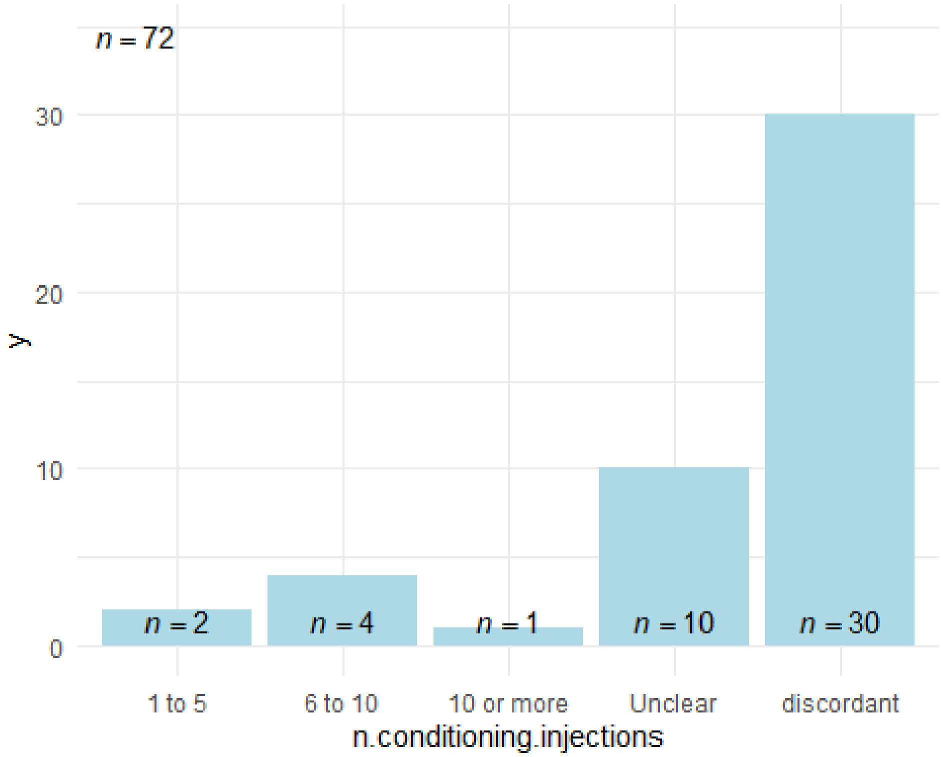

Figure s25. Question 23: Pooled QC samples were reported to have been injected ________. (choose the best answer)

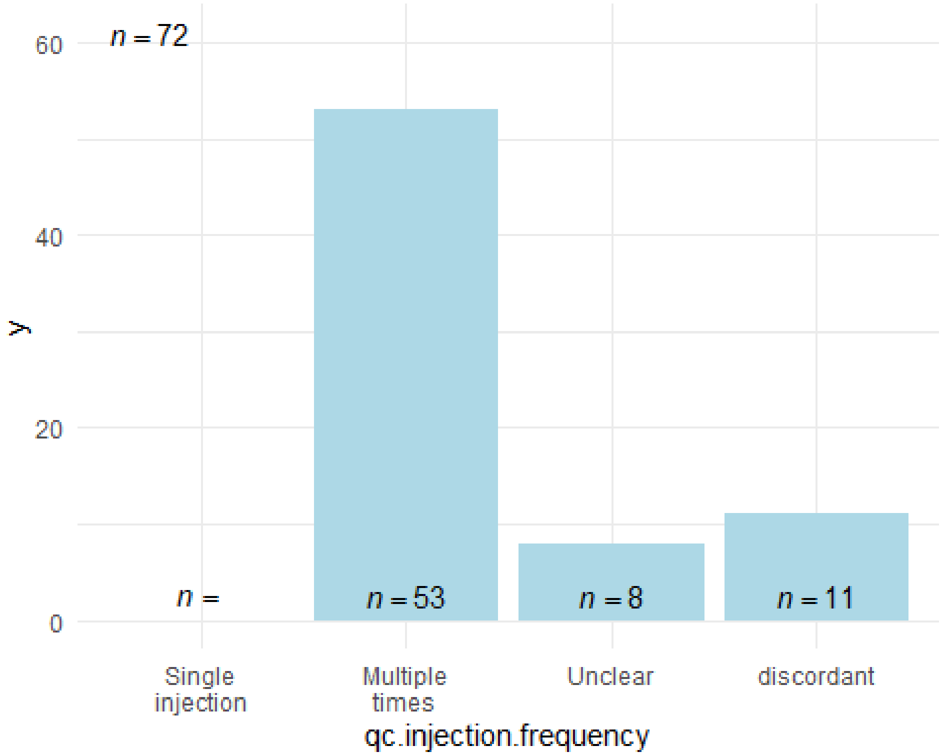

Figure s26. Question 24: Multiple QC injections were reported to have been made from _____.

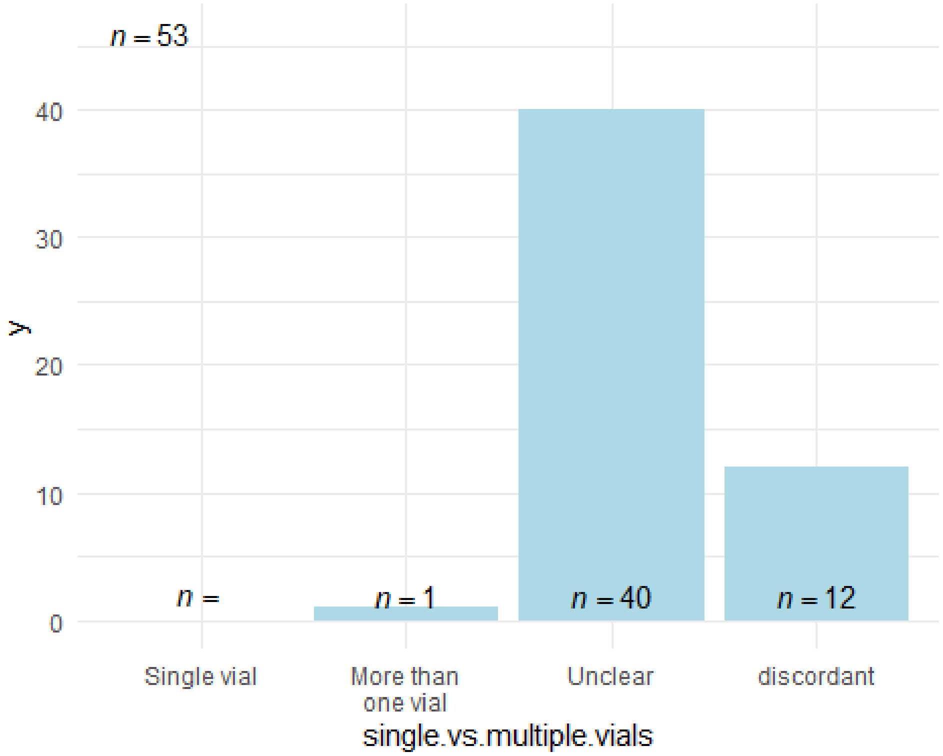

Figure s27. Question 25: At which position within the batch were QC samples reported to have been injected _____________? (choose all that apply) At the beginning of the batch

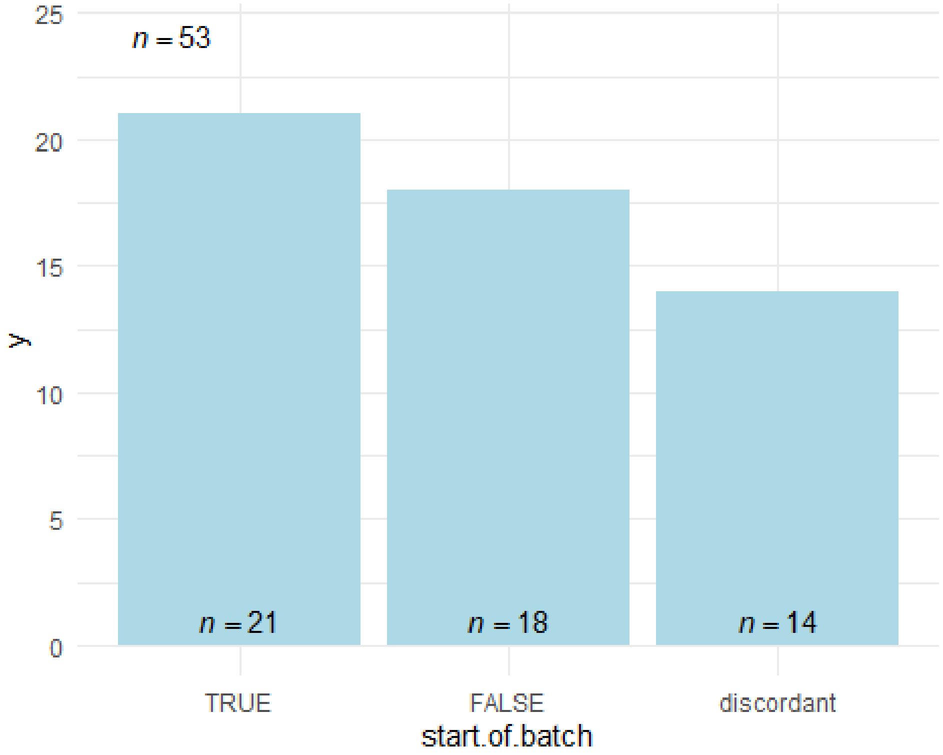

Figure s28. Question 26: At which position within the batch were QC samples reported to have been injected ______________? (choose all that apply) At the end of the batch

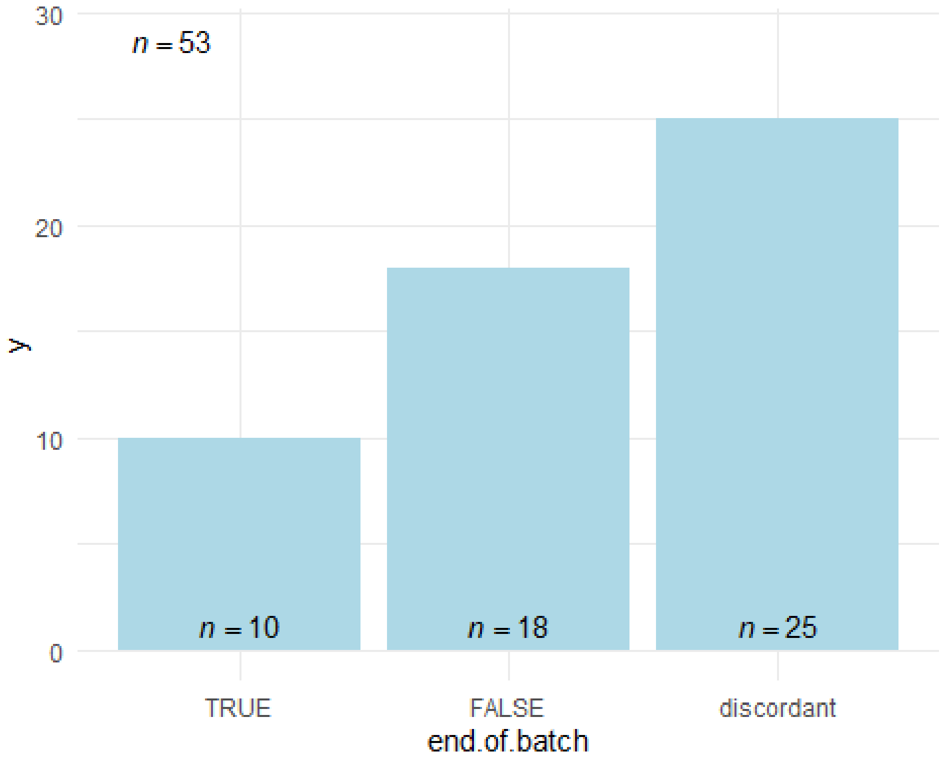

Figure s29. Question 27: At which position within the batch were QC samples reported to have been injected _____________? (choose all that apply) In the middle of a batch

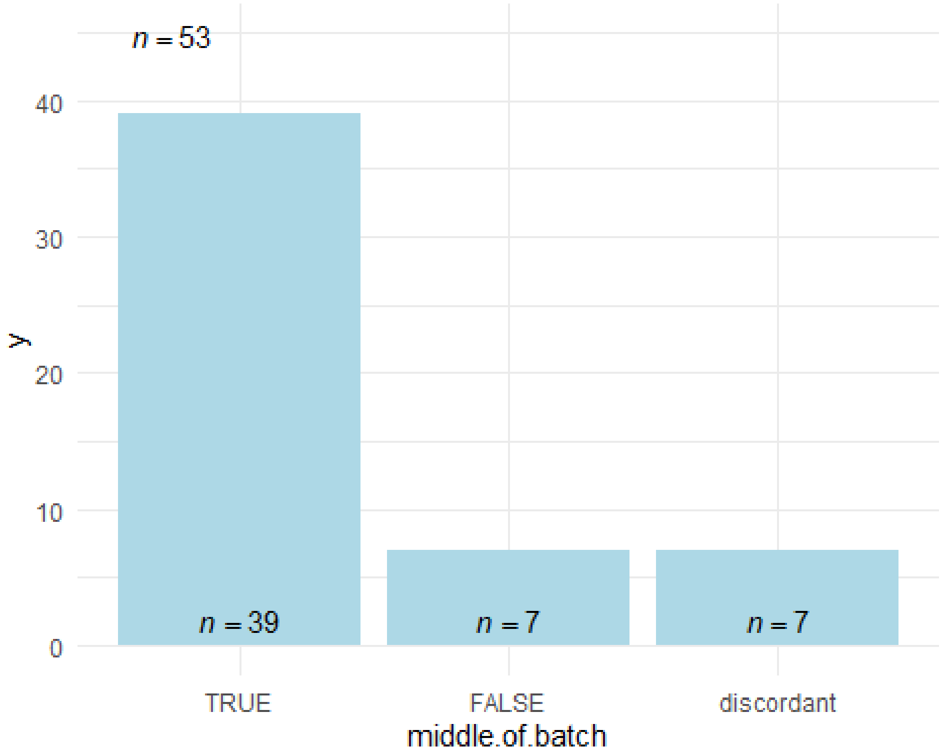

Figure s30. Question 29: At which position within the batch were QC samples reported to have been injected ______________? (choose all that apply) Unclear

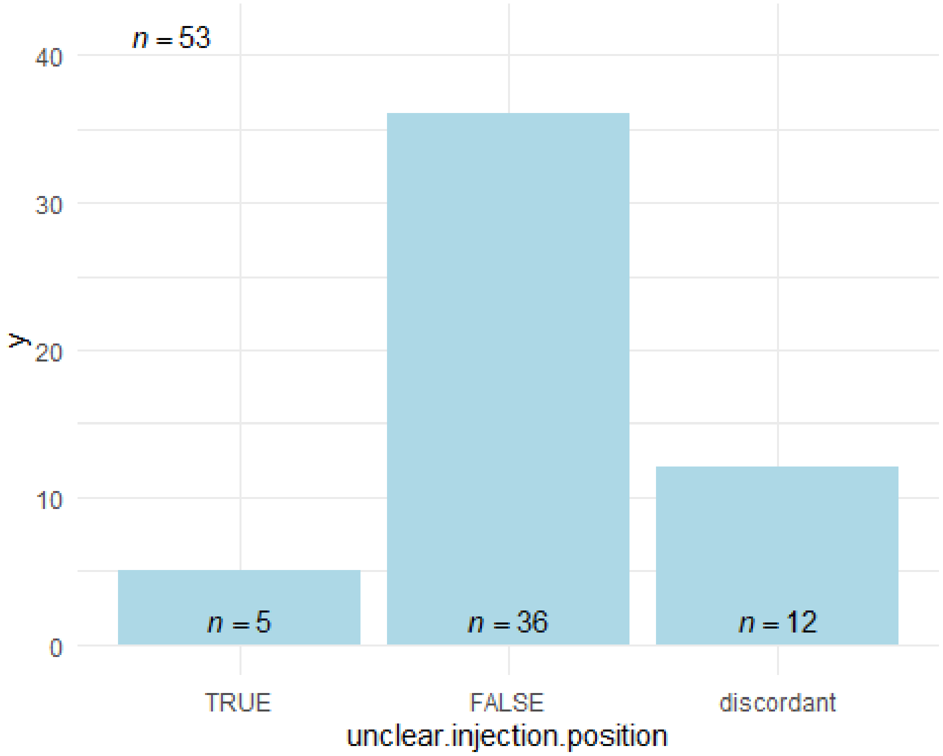

Figure s31. Question 30: At what frequency were QC samples reported to have been injected?

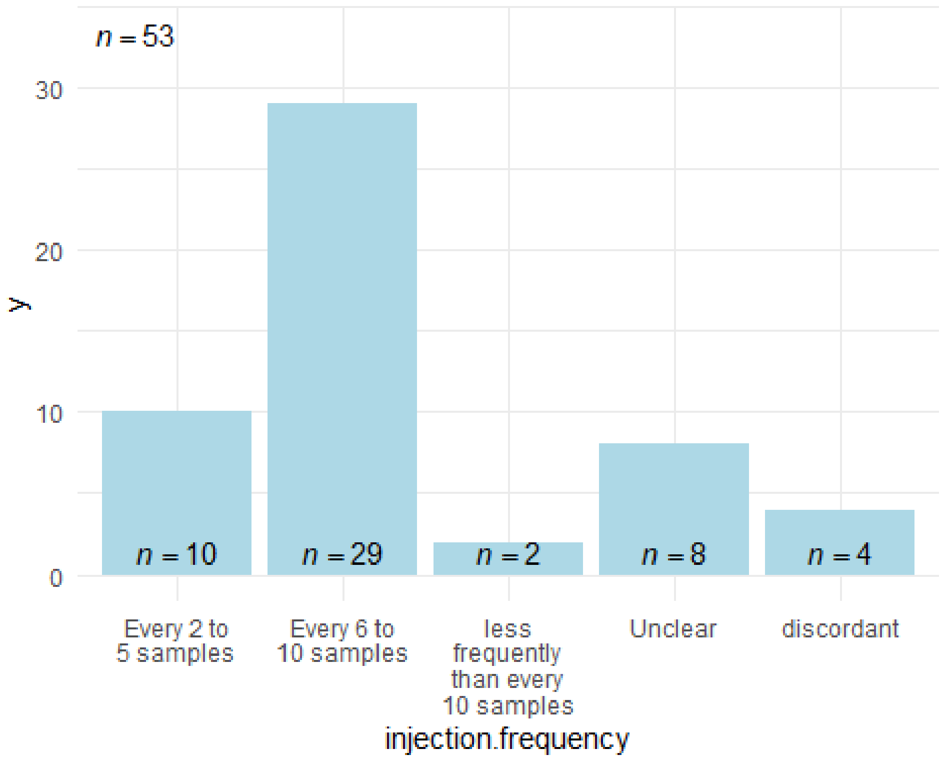

Figure s32. Question 31: Was a dilution series of the pooled QC sample reported to have been used? (choose the best answer)

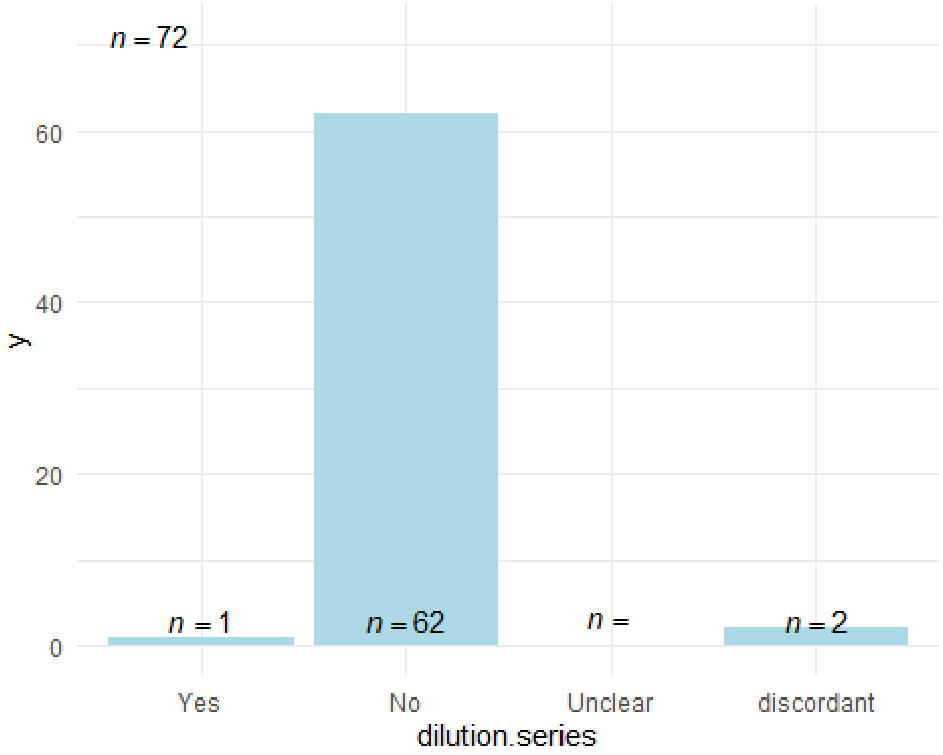

Figure s33. Question 32: Which criterion was reported to have been used with a pooled QC sample to filter features with low precision? (choose the best answer)

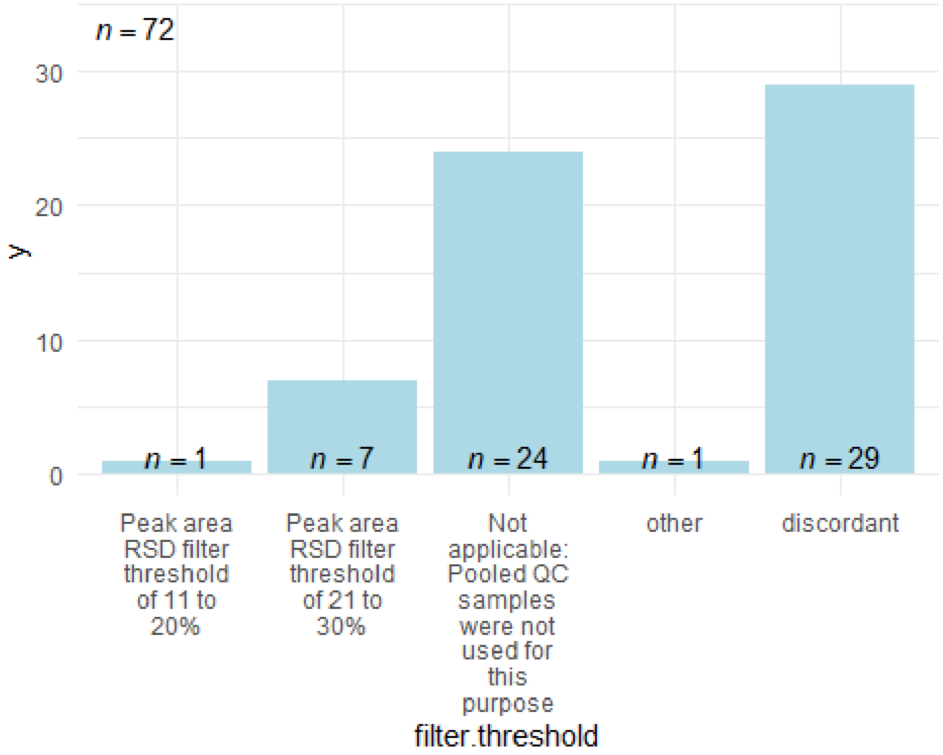

Figure s34. Question 33: Were pooled QC samples used in PCA analysis?

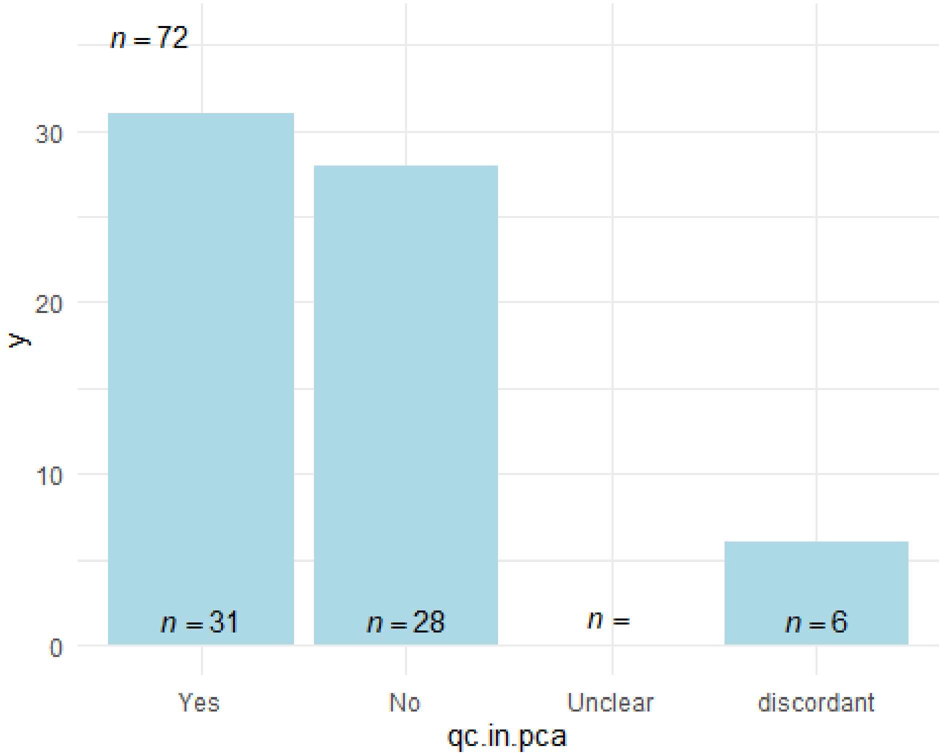

Figure s35. Question 34: If PCA was reported to have been used to evaluate pooled QC clustering/variability in a study, the analysis included ______. (choose the best answer)

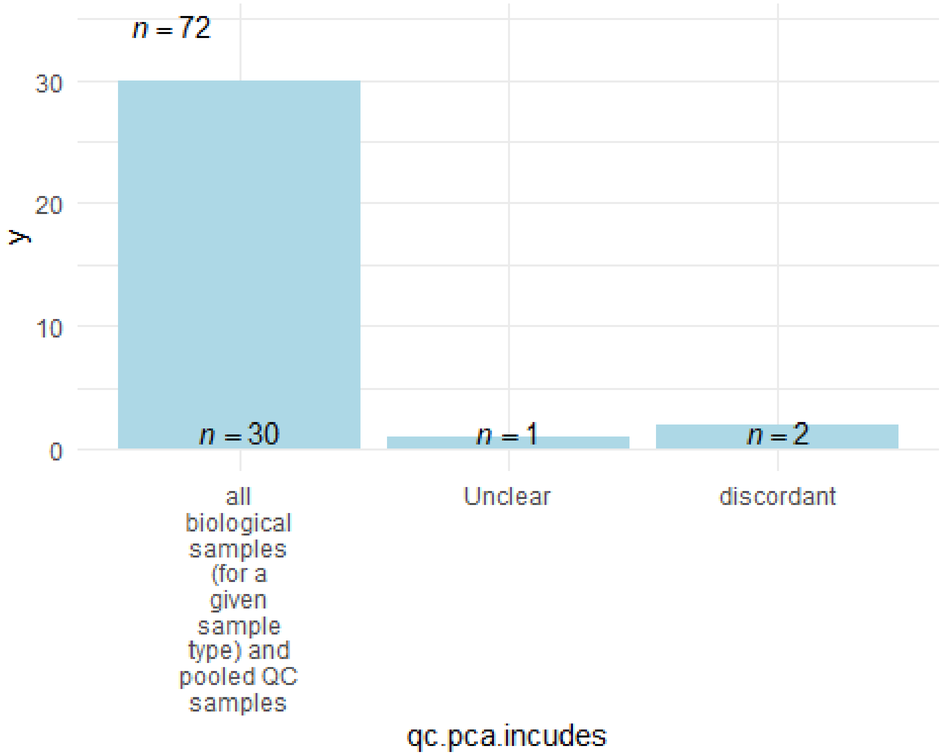

Figure s36. Question 35: What criteria were reported to have been used to evaluate pooled QC clustering/variability in the PCA plot? (choose the best answer)

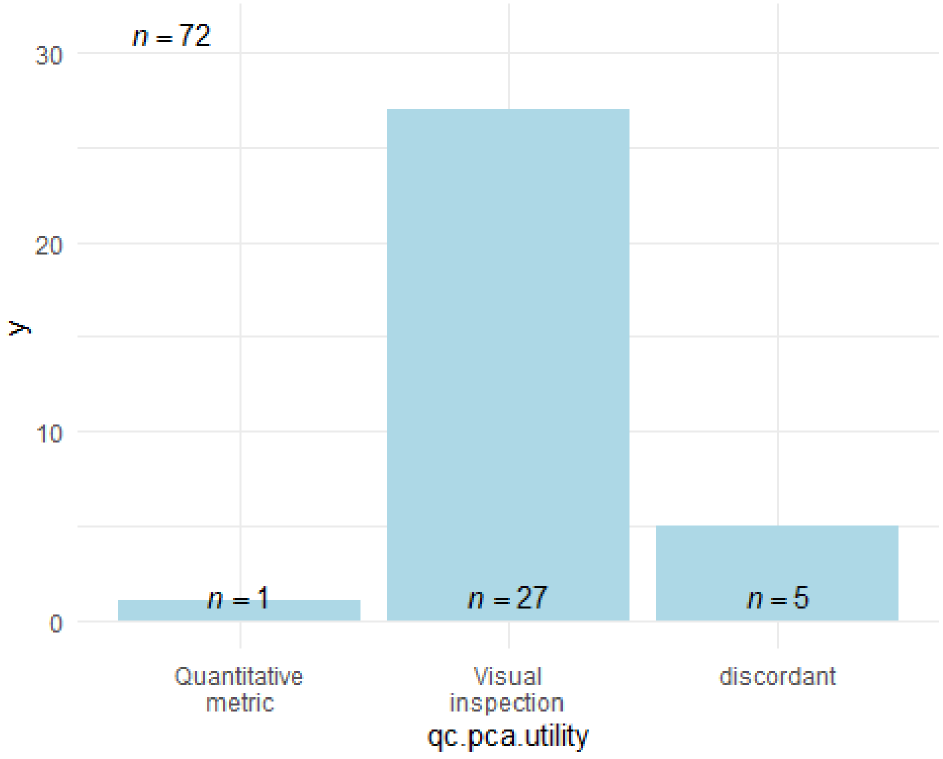

Figure s37. Question 36: Was the same QC approach used for all matrices

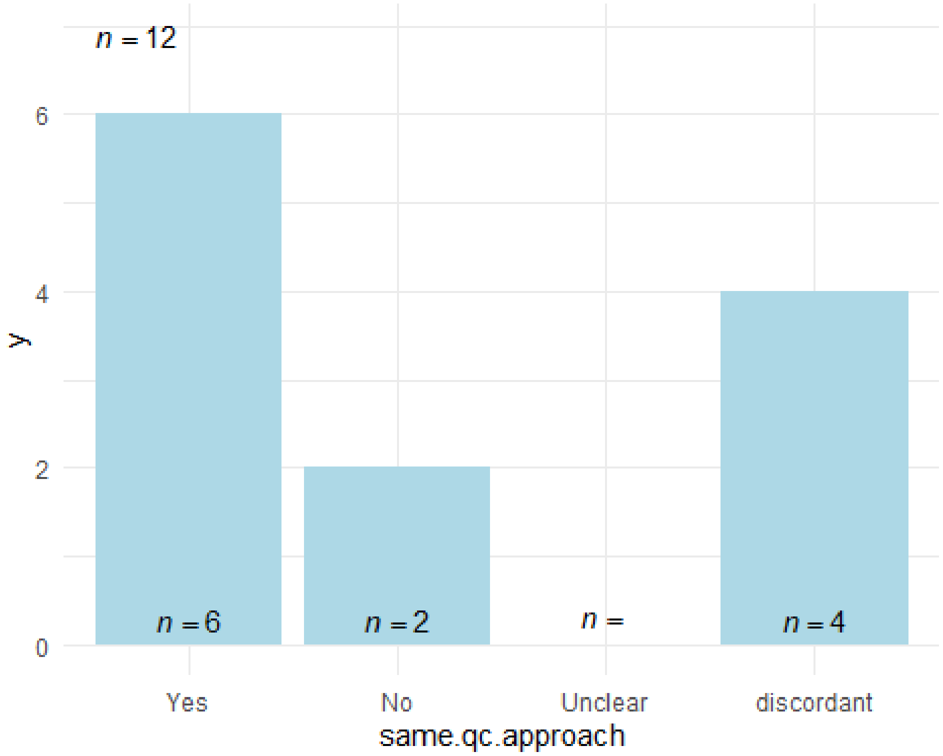

Results: Other QC Approaches

In the section below, questions about other quality control approaches are addressed. These questions are not strictly dependent on whether a pooled QC sample was used, thus the full dataset is again considered.

Figure s38. Question 37: Which - if any - alternate QC samples/approaches were reported to have been utilized in this study? (Choose all that apply) Internal Standards

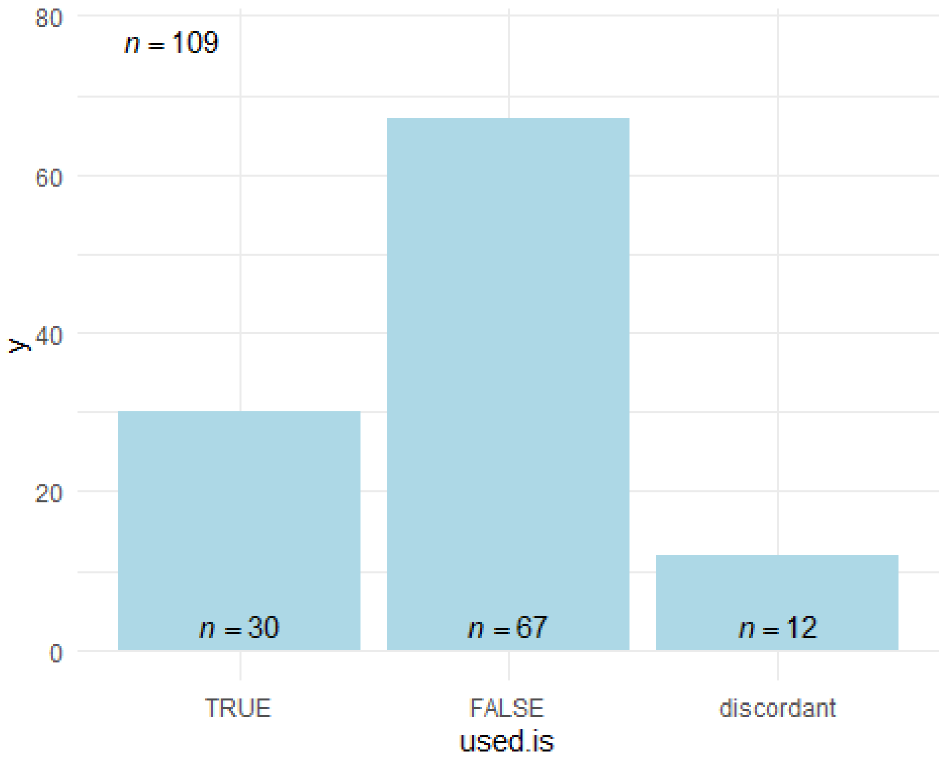

Figure s39. Question 38: Which - if any - alternate QC samples/approaches were reported to have been utilized in this study? (Choose all that apply) Long Term Reference QC sample or Intralaboratory QC sample

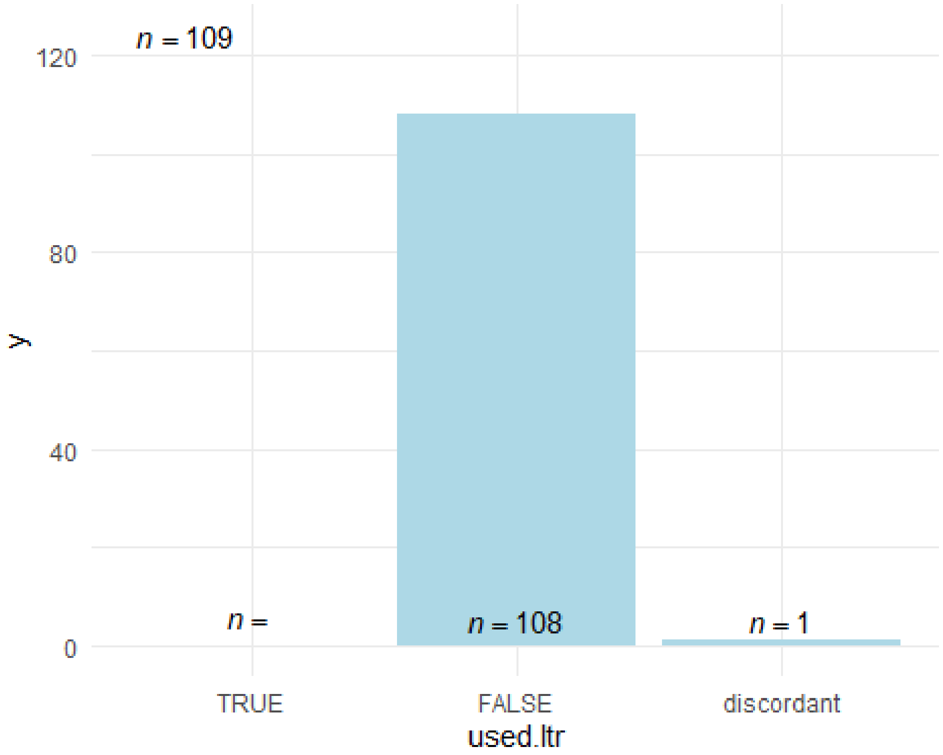

Figure s40. Question 39: Which - if any - alternate QC samples/approaches were reported to have been utilized in this study? (Choose all that apply) Multiple injections of each experimental sample

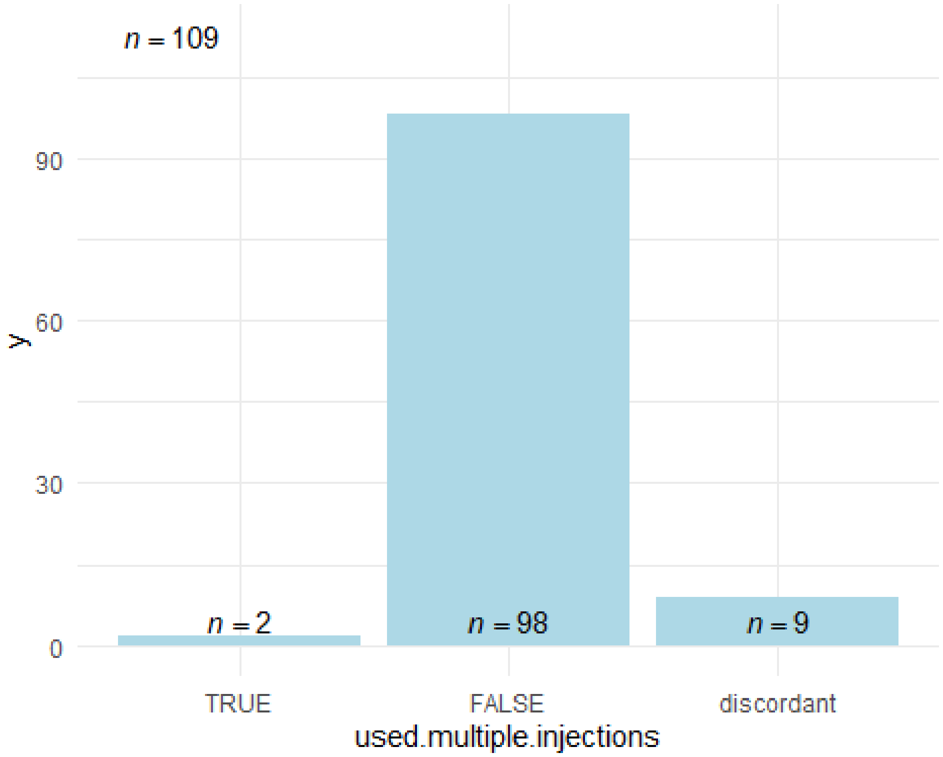

Figure s41. Question 40: Which - if any - alternate QC samples/approaches were reported to have been utilized in this study? (Choose all that apply) No other QC sample types were used

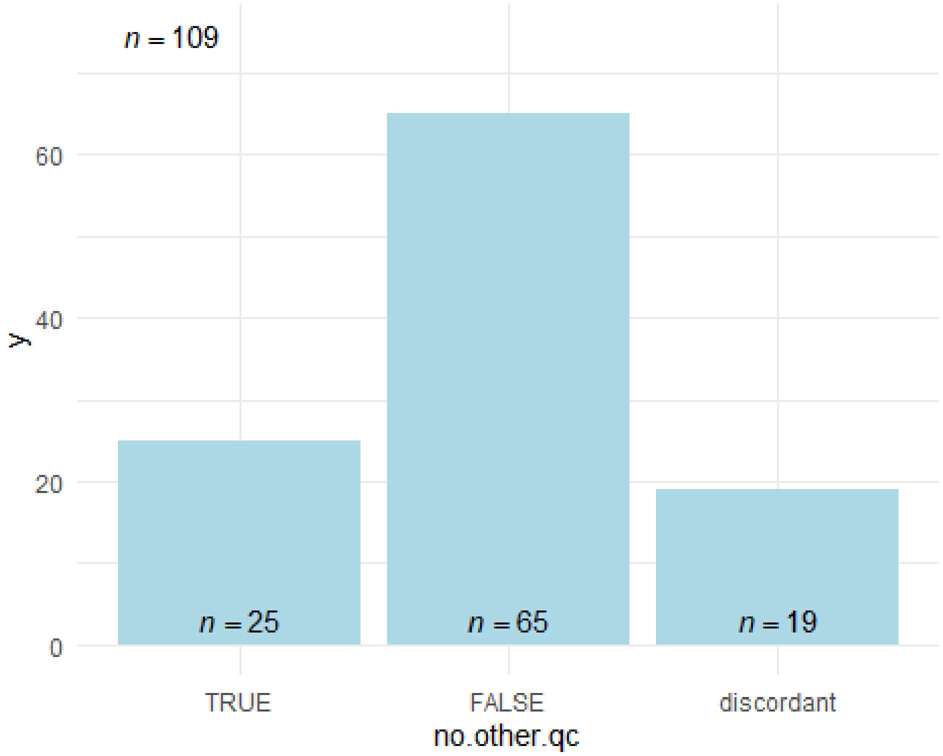

Figure s42. Question 41: Which - if any - alternate QC samples/approaches were reported to have been utilized in this study? (Choose all that apply) Other

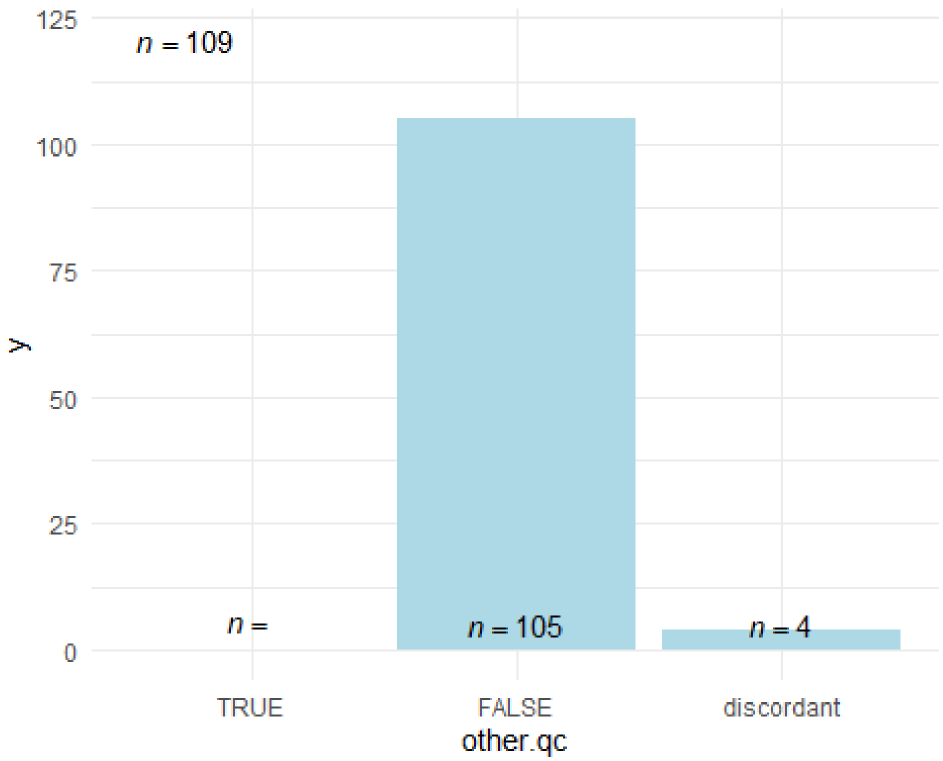

Figure s43. Question 42: Which - if any - alternate QC samples/approaches were reported to have been utilized in this study? (Choose all that apply) Standard Reference Material or Interlaboratory QC sample

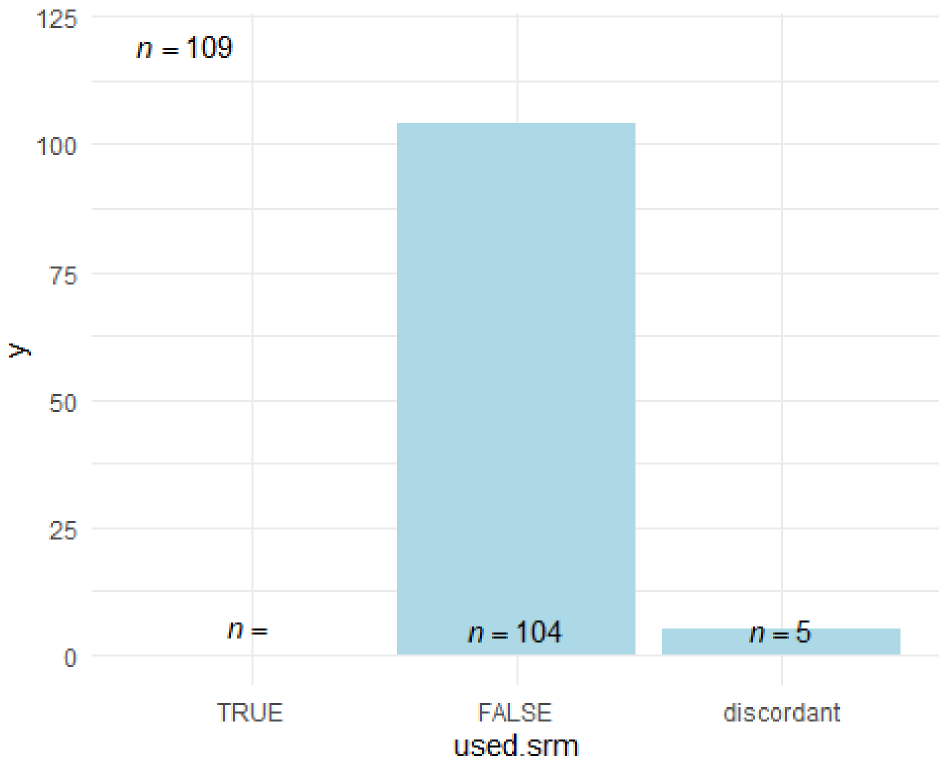

Figure s44. Question 44: Which - if any - alternate QC samples/approaches were reported to have been utilized in this study? (Choose all that apply) Blanks (system; extraction; solvent; etc)

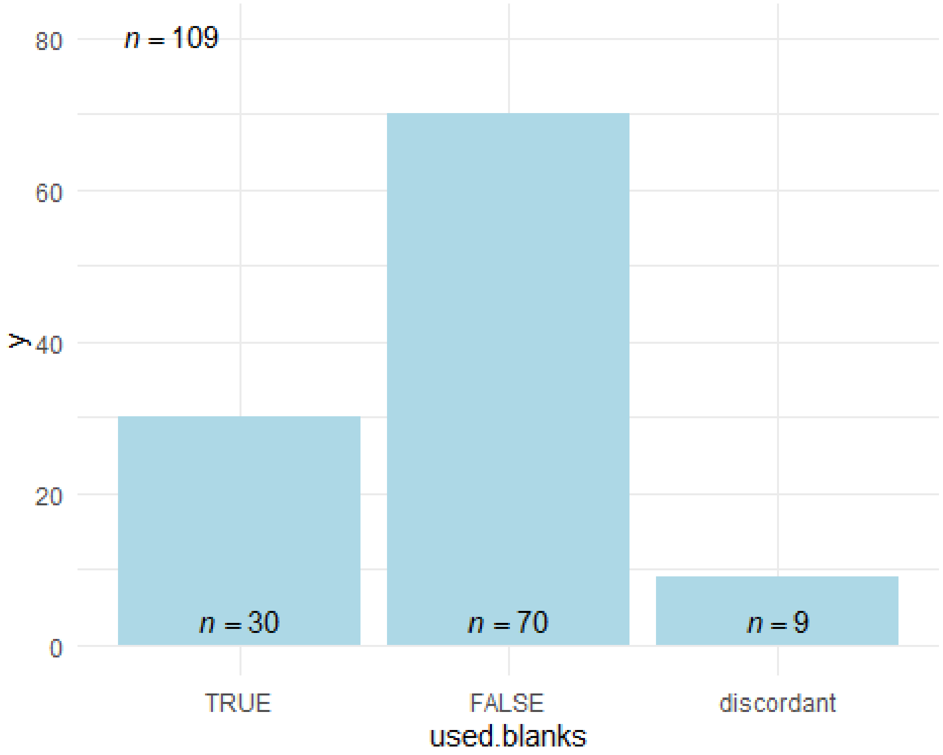

Figure s45. Question 45: Which - if any - alternate QC samples/approaches were reported to have been utilized in this study? (Choose all that apply) System suitability sample (i.e. pubmed pid 29805336)

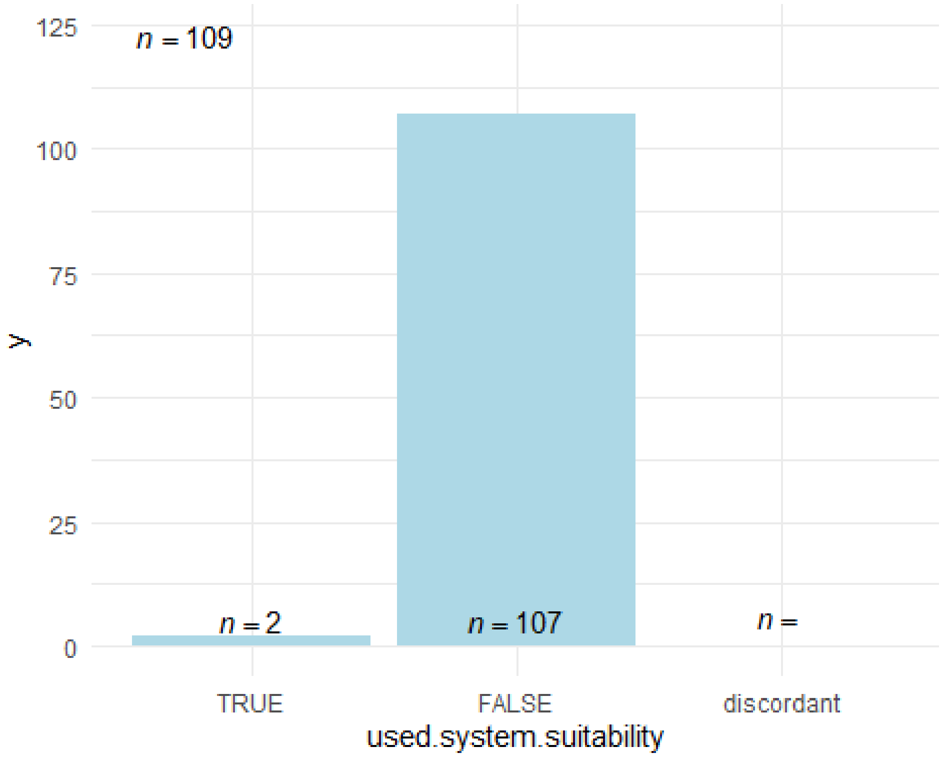

Figure s46. Question 46: Did this manuscript use citations to previous studies in describing their quality control methods? Answer 'Yes' if the reference suggests there are more details on quality control methods in the referenced paper and 'No' if citations were not used or if the reference suggests it is not related to quality control approach.

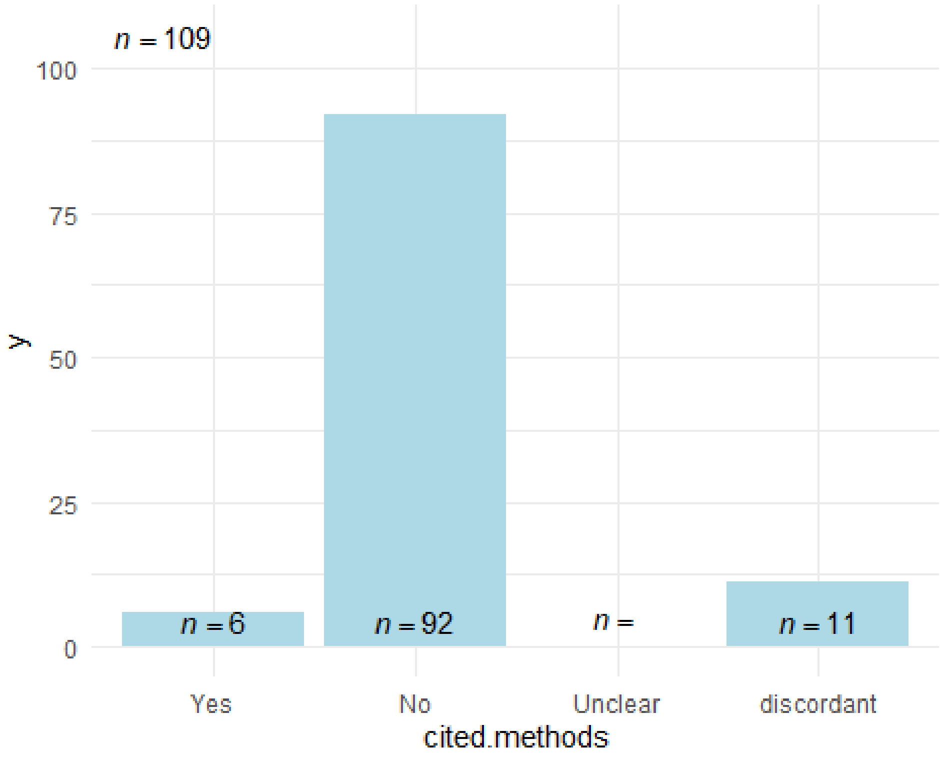

Figure s47. Question 47: The untargeted work was reported to have been performed at ______. (choose all that apply) Commercial metabolomics provider

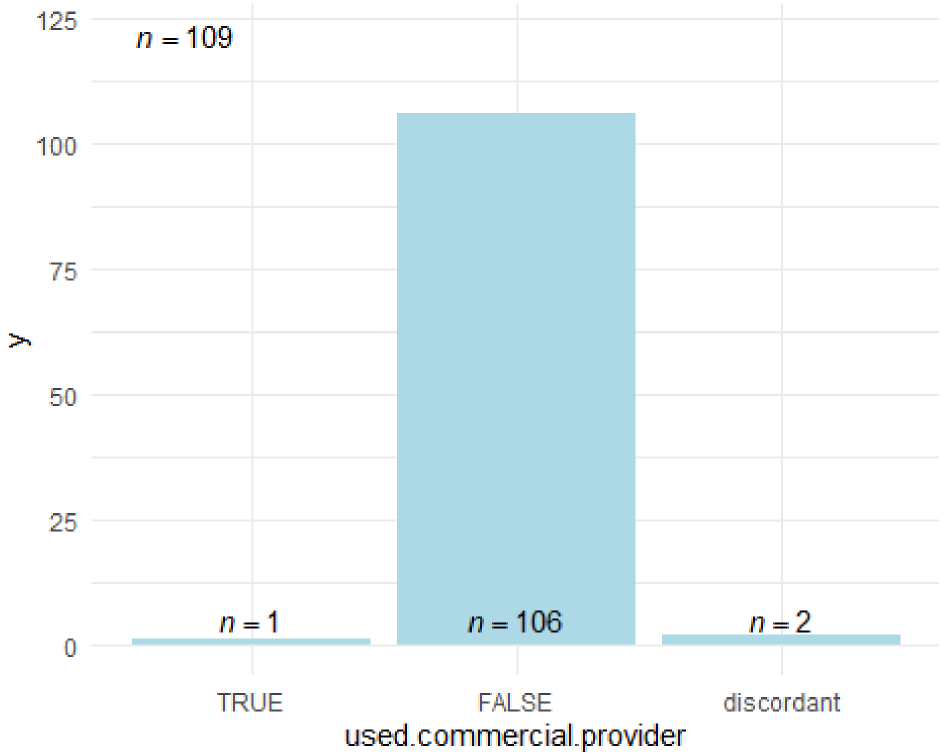

Figure s48. Question 48: The untargeted work was reported to have been performed at ______. (choose all that apply) Core metabolomics facility

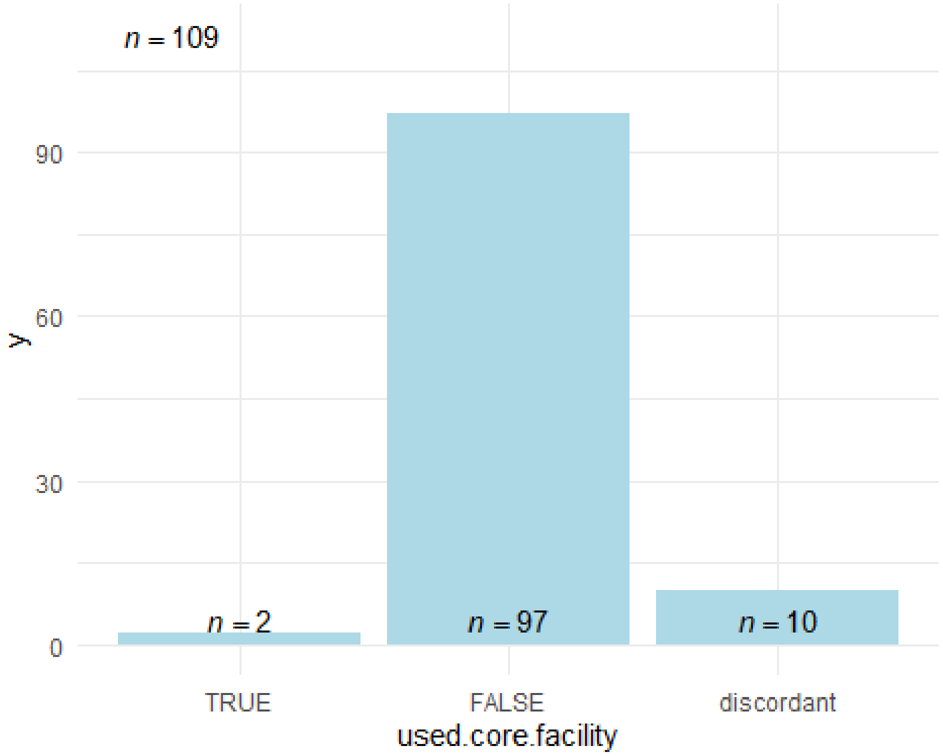

Figure s49. Question 49: The untargeted work was reported to have been performed at ______. (choose all that apply) Independent invesitigator laboratory

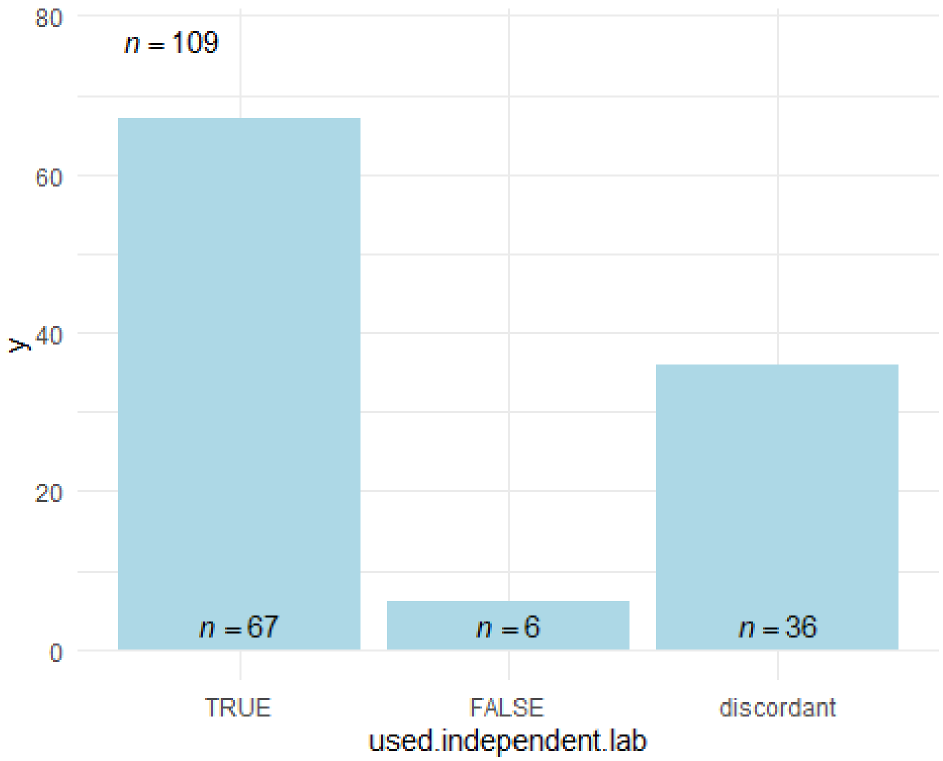

Publication Figures:

a.

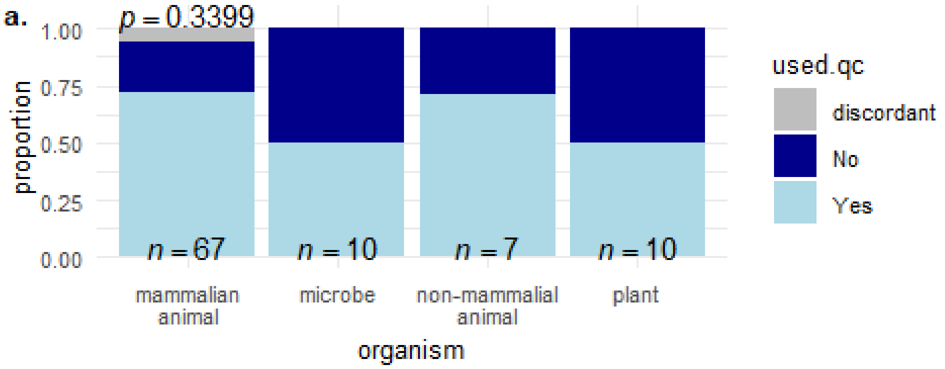

b.

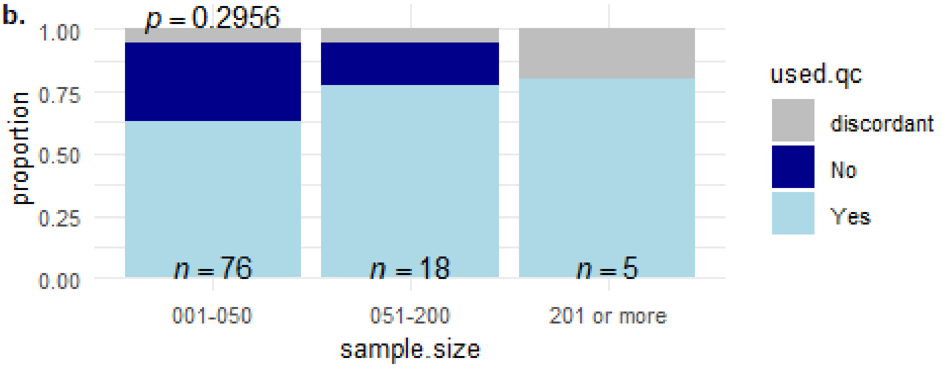

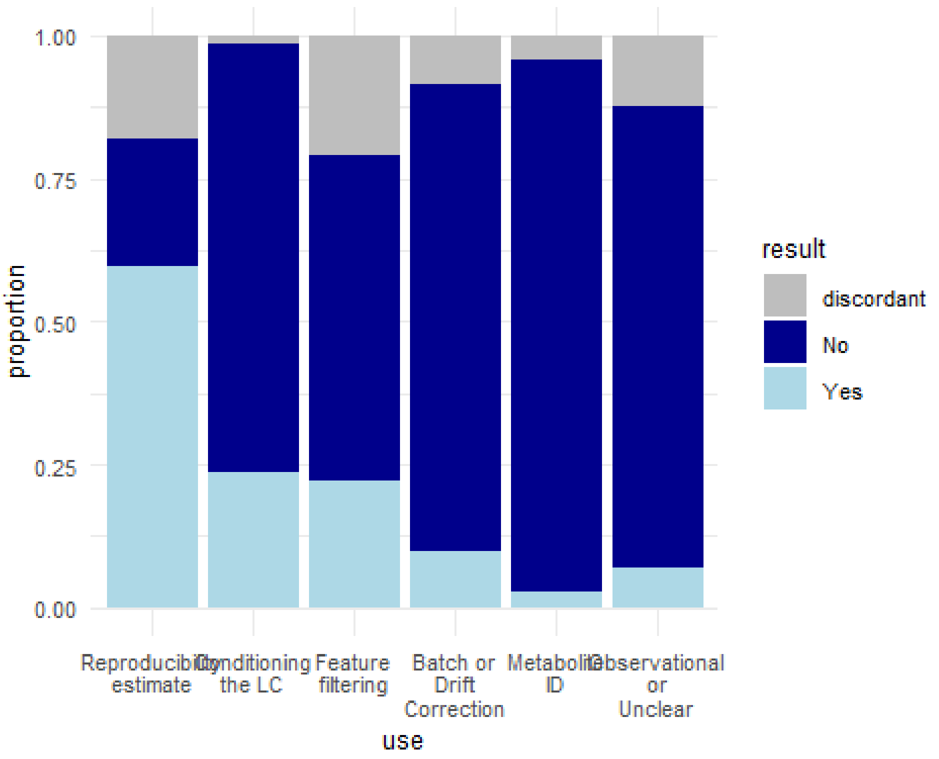

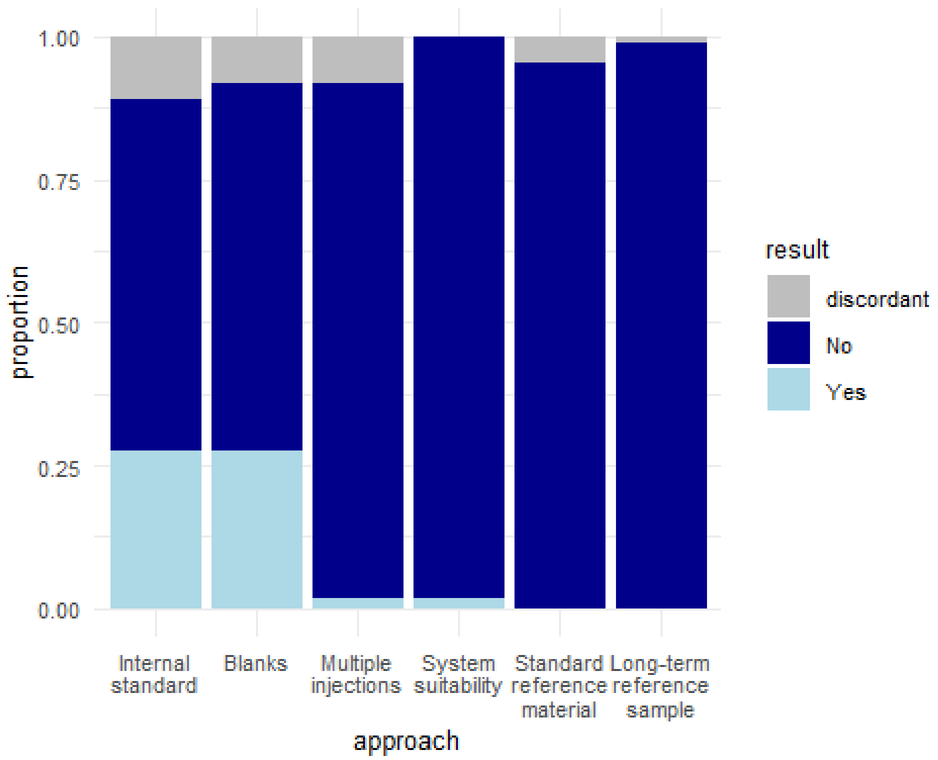
1.00
0.75
0.50
0.25
0.00
proportion
Internal standard
Blanks
Multiple injections
System suitability
Standard reference material
Long-term reference sample
approach
result
discordant
No
Yes
