# Supplementary material for: Current Practices in LC-MS Untargeted Metabolomics: A Scoping Review on the Use of Pooled Quality Control Samples
Source: Anal Chem. 2023 Dec 6;95(51):18645–54. doi: 10.1021/acs.analchem.3c02924 (PMC10753522; doi:10.1021/acs.analchem.3c02924)
Supplement: Supplementary file 1 — ac3c02924_si_001.zip [file ac3c02924_si_001.zip › SupportingInformation.docx]

**Supporting Information:**

Current practices in LC-MS untargeted metabolomics: a scoping review on the use of pooled quality control samples.

Broeckling, Corey D.*^,a^; Beger, Richard D.^b^; Cheng, Leo L.^c^; Cumeras, Raquel^d^; Cuthbertson, Daniel^e^; Dasari, Surendra^f^; Davis, W. Clay^g^; Dunn, Warwick B.^h^; Evans, Anne M.^i^; Fernández-Ochoa^j^, Alvaro; Gika, Helen^k^; Goodacre, Royston^l^; Goodman, Kelli D^m^; Gouveia, Goncalo J.^n^; Hsu, Ping-Ching^o^; Kirwan, Jennifer A^p^; Kodra, Dritan^q^; Kuligowski, Julia^r^; Lan, Renny S^s^.; Monge, María Eugenia^t^; Moussa, Laura W^u^; Nair, Sindhu^v^; Reisdorph, Nichole^w^; Sherrod, Stacy D.^x^; Ulmer, Candice Z^y^; Vuckovic, Dajana^z^; Yu, Li-Rong^aa^; Zhang, Bo^bb^; Theodoridis, Georgios*^,cc^; Mosley, Jonathan D*^,dd^; on behalf of the metabolomics Quality Assurance and Quality Control Consortium (mQACC)

*cbroeckl@colostate.edu, gtheodor@chem.auth.gr, mosley.jonathan@epa.gov

**Affiliations:**

1. Broeckling, Corey D.  Analytical Resources Core: Bioanalysis and Omics Center; Department of Agricultural Biology, Colorado State University, Fort Collins, CO, 80525.  [corey.broeckling@colostate.edu](mailto:corey.broeckling@colostate.edu).  970-491-2273.
2. Beger, Richard D. Division of Systems Biology, National Center for Toxicological Research, Jefferson, AR, 72079, Richard.Beger@fda.hhs.gov 870 543-7080. ORCID ID: 0000-0003-4380-2356
3. Cheng, Leo L. Departments of Radiology and Pathology, Massachusetts General Hospital, Harvard Medical School, Boston, Massachusetts 02114. [lcheng@mgh.harvard.edu](mailto:lcheng@mgh.harvard.edu) 617-724-6593
4. Cumeras, Raquel. Oncology Department, Nutrition and Metabolism Department, Institut d’Investigació Sanitària Pere Virgili (IISPV), Univeristat Rovira i Virgili (URV), c/Escorxador s/n, 43003, Tarragona, Spain. https://orcid.org/0000-0003-4663-4247 [raquel.cumeras@iispv.cat](mailto:raquel.cumeras@iispv.cat)
5. Cuthbertson, Daniel J.  Agilent Technologies Inc., 5301 Stevens Creek Blvd, Santa Clara, CA 95051, USA [daniel_cuthbertson@agilent.com](mailto:daniel_cuthbertson@agilent.com)
6. Dasari, Surendra. Department of Quantitative Health Sciences, Mayo Clinic, Rochester, MN, dasari.surendra@mayo.edu
7. Davis, W. Clay. National Institute of Standards and Technology, Chemical Sciences Division, 331 Fort Johnson Road, Charleston, SC 29412. clay.davis@nist.gov. <https://orcid.org/0000-0001-9076-2620>
8. Dunn, Warwick B.  Centre for Metabolomics Research, Department of Biochemistry and Systems Biology, Institute of Systems, Molecular and Integrative Biology, University of Liverpool, BioSciences Building, Crown St., Liverpool, UK, L69 7ZB. <https://orcid.org/0000-0001-6924-0027>. [warwick.dunn@liverpool.ac.uk](mailto:warwick.dunn@liverpool.ac.uk)
9. Evans, Anne M. Metabolon, Inc. 617 Davis Drive, Suite 100, Morrisville, NC, 27560, USA. <https://orcid.org/0000-0002-0827-276X>. aevans@metabolon.com
10. Fernández-Ochoa, Álvaro. Department of Analytical Chemistry, University of Granada, Granada, Spain. [alvaroferochoa@ugr.es](mailto:alvaroferochoa@ugr.es)
11. Goodman, Kelli D. Metabolon, Inc. 617 Davis Drive, Suite 100, Morrisville, NC 27560, USA. [kgoodman@metabolon.com](mailto:kgoodman@metabolon.com).
12. Gika, Helen  School of Medicine, Aristotle University of Thessaloniki, 54124 Thessaloniki, Greece, [gkikae@auth.gr](mailto:gkikae@auth.gr)
13. Goodacre, Royston Centre for Metabolomics Research, Department of Biochemistry and Systems Biology, Institute of Systems, Molecular and Integrative Biology, University of Liverpool, BioSciences Building, Crown St., Liverpool, UK, L69 7ZB. ORCID ID: 0000-0003-2230-645X [roy.goodacre@liverpool.ac.uk](mailto:roy.goodacre@liverpool.ac.uk)
14. Gouveia, Goncalo J., Institute for Bioscience and Biotechnology Research, National Institute of Standards and Technology, University of Maryland, Gudelsky Drive, Rockville, Maryland, 20850, USA, goncalog@uga.edu
15. Hsu, Ping-Ching. Department of Environmental Health Sciences, University of Arkansas for Medical Sciences, Little Rock, AR, 72205-7190. PHsu@uams.edu. 501-526-6687. ORCID ID: 0000-0002-1749-0662
16. Kirwan Jennifer A. Max Delbrück Center Robert Rössle Strasse 10 13125 Berlin. [jennifer.kirwan@bih-charite.de](mailto:jennifer.kirwan@bih-charite.de)
17. Kodra, Dritan, Department of Chemistry, Aristotle University of Thessaloniki; [drity.kodra@gmail.com](mailto:drity.kodra@gmail.com)
18. Kuligowski, Julia. Neonatal Research Group, Health Research Institute La Fe, Avenida Fernando Abril Martorell 106, 46026 Valencia, Spain. [julia.kuligowski@uv.es](mailto:julia.kuligowski@uv.es)
19. Lan. Renny S. Arkansas Children’s Nutrition Center, Little Rock, AR, 72202-3591, USA. [Slan@uams.edu](mailto:Slan@uams.edu) ORC ID: 0000-0001-8346-043X
20. Monge, María Eugenia. Centro de Investigaciones en Bionanociencias (CIBION), Consejo Nacional de Investigaciones Científicas y Técnicas (CONICET), Godoy Cruz 2390, C1425FQD, Ciudad de Buenos Aires, Argentina. [mslab.cibion@gmail.com](mailto:mslab.cibion@gmail.com) ORCID ID: 0000-0001-6517-5301
21. Moussa, Laura. Center for Veterinary Medicine, Office of New Animal Drug Evaluation, U.S. Food and Drug Administration. [Laura.Moussa@fda.hhs.gov](mailto:Laura.Moussa@fda.hhs.gov). This article reflects the views of the author and should not be construed to represent FDA’s views or policies.
22. Nair, Sindhu:  Department of Biological Sciences, University of Alberta, Edmonton, AB T6G 2G2 Canada  [snair@ualberta.ca](mailto:snair@ualberta.ca)
23. Reisdorph, Nichole.  Department of Pharmaceutical Sciences, University of Colorado-Anschutz Medical Campus, Aurora, CO, USA. [NICHOLE.REISDORPH@CUANSCHUTZ.EDU](mailto:NICHOLE.REISDORPH@CUANSCHUTZ.EDU)
24. Sherrod, Stacy D., Department of Chemistry and Center for Innovative Technology, Vanderbilt University, Nashville, TN, USA [stacy.d.sherrod@vanderbilt.edu](mailto:stacy.d.sherrod@vanderbilt.edu); ORCID ID: 0000-0002-2346-230X
25. Ulmer Holland, Candice Z. Chemistry Branch, Eastern Laboratory, Office of Public Health Science, USDA-FSIS. Athens, GA, USA [candice.ulmer@usda.gov](mailto:candice.ulmer@usda.gov)
26. Vuckovic, Dajana, Department of Chemistry and Biochemistry, Concordia University, 7141 Sherbrooke Street West, Montreal, QC H4B 1R6, Canada [dajana.vuckovic@concordia.ca](mailto:dajana.vuckovic@concordia.ca) 0000-0002-7764-8492
27. Yu, Li-Rong. Division of Systems Biology, National Center for Toxicological Research, U.S. Food and Drug Administration, Jefferson, AR 72079, USA. Lirong.Yu@fda.hhs.gov. 870 543-7052. ORCID ID: 0000-0001-6084-991X
28. Zhang, Bo, Olaris, Inc., 175 Crossing Blvd Suite 410, Framingham, MA 01702, USA [bozhangchem@gmail.com](mailto:bozhangchem@gmail.com)
29. Theodoridis Georgios, Department of Chemistry, Aristotle University of Thessaloniki, 54124 Greece; [gtheodor@chem.auth.gr](mailto:gtheodor@chem.auth.gr)
30. Mosley, Jonathan D. Center for Environmental Measurement and Modeling, Environmental Protection Agency, Athens, Georgia 30605, United States. [mosley.jonathan@epa.gov](mailto:mosley.jonathan@epa.gov) ORCID ID: 0000-0002-9300-6924

[Supporting Documents: 3](#_Toc148694695)

[Supplemental_1_literature.surveyed.xlsx: 3](#_Toc148694696)

[Supplemental_2_fullSurvey.pdf: 3](#_Toc148694697)

[Supplemental_3_individual.responses.xlsx: 3](#_Toc148694698)

[Supplemental_4_analysisRmarkdown.Rmd: 3](#_Toc148694699)

[Supplemental_5_detailed.output.pdf: 3](#_Toc148694700)

# Supporting Documents:

Supplemental_1_literature.surveyed.xlsx:

- A tabular description of the literature surveyed for this study. Also provided as .csv format for computer readability

Supplemental_2_fullSurvey.pdf:

- The full survey including all questions and answers, as used in this study.

Supplemental_3_individual.responses.xlsx:

- Each individual response from each respondent. Reviewers are anonymized.

Supplemental_4_analysisRmarkdown.Rmd:

- Rmarkdown document used to perform all filtering, data analysis, and figure creation.

Supplemental_5_detailed.output.pdf:

- PDF output from supplemental_4_analysisRmarkdown.Rmd.
